# Characteristics of culture-condition stimulated exosomes or their loaded hydrogels in comparison with other extracellular vesicles or MSC lysates

**DOI:** 10.3389/fbioe.2022.1016833

**Published:** 2022-09-16

**Authors:** Yu Luo, Zhihua Li, Xinxin Wang, Juan Wang, Xingxiang Duan, Ruohan Li, Youjian Peng, Qingsong Ye, Yan He

**Affiliations:** ^1^ Center of Regenerative Medicine and Department of Stomatology, Renmin Hospital of Wuhan University, Wuhan, China; ^2^ Department of Orthodontics, School and Hospital of Stomatology, Nanchang University, Nanchang, China; ^3^ Institute of Regenerative and Translational Medicine, Tianyou Hospital, Wuhan University of Science and Technology, Wuhan, China

**Keywords:** extracellular vesicles, exosomes, exosomes loaded hydrogels, microvesicles, apoptotic bodies, mesenchymal stem cell lysates

## Abstract

Recently, it has become popular to study the use of extracellular vesicles (EVs) secreted by stem cells to repair damaged tissues or lost cells. Various cell types and physiological fluids release EVs, and they play an important role in cell-to-cell communication. Moreover, EVs have been implicated in important processes, such as immune responses, homeostasis maintenance, coagulation, inflammation, cancer progression, angiogenesis, and antigen presentation. Thus, EVs participate in both physiological and pathological progression. The main classes of EVs include exosomes, microvesicles (MVs), and apoptotic bodies (ApoBDs). Exosomes, which carry a mass of signal molecules such as RNA, DNA, proteins, and lipids, are the most important of these EVs subsets. Currently, exosomes are generating substantial interest in the scientific community. Exosomes loaded hydrogels or under different cultural environments exhibit different properties and functions. Therefore, the exosomes obtained from different sources and conditions are worth reviewing. More importantly, no review article has compared the different EVs, such as exosomes, MVs, ApoBDs, and mesenchymal stem cell (MSC) lysates, which are special soluble substances. The differentiation between EVs and MSC lysates is a logical approach. Accordingly, this review provides an update on the latest progress in studying the roles of culture-condition stimulated exosomes or their loaded hydrogels and the differentiation between exosomes, MVs, ApoBDs, and MSC lysates. Published studies were retrieved from the PubMed® database for review.

## 1 Introduction

A large body of experimental and clinical studies revealed that most MSC-based therapies’ success is attributable to the paracrine secretome, which consists of soluble components and encapsulated extracellular vesicles (EVs) (Lee and Kim, 2021). The secreted EVs, which were recognized in 1983, are predominantly endosomal in origin and contain a cargo of miRNA, mRNA, and proteins (Keshtkar et al., 2018), moreover, they are key factors in cell-cell communication, tissue homeostasis, cell differentiation, organ development and remodeling (Gurung et al., 2021). EVs comprise a wide spectrum of vesicles with a diameter ranging from 8 nm to several microns, in the following categories: 1) nanovesicles (8–12 nm), which have an unknown origin. 2) exosomes (30–150 nm), which are released *via* multivesicular bodies (MVB) fusion with the plasma membrane. 3) MVs (200–1,000 nm), which result from direct budding from the plasma membrane. 4) ApoBDs (1,000–5,000 nm), which are randomly released by plasma membrane blebs containing cytoplasm and organelle fragments. 5) large oncosomes (1–10 μm), which also result from budding from the plasma membrane (Sedgwick and D'Souza-Schorey, 2018).

Exosomes which exhibit a diameter ranging from 30 to 150 nm and a density of 1.13–1.19 g/ml in a sucrose gradient are a main subclass of EVs, they can be collected by centrifugation at 100,000 × g. After isolation, exosomes could be stored without toxic cryoprotectant agents at −80°C for more than 6 months while maintaining their functions (Ma et al., 2020; Tang et al., 2021). The biogenesis of exosomes occurs *via* the endocytosis–exocytosis pathway when cells absorb small amounts of intracellular fluid in a specific membrane region and sprout as early endosomes. They begin to mature and expand into late endosomes, followed by the formation of intraluminal vesicles (ILVs) or MVBs in the luminal space of late endosomes (Nikfarjam et al., 2020). MVBs then fuse with the cell membrane and are released into the extracellular environment, at which point the vesicles are termed exosomes (Rezaie et al., 2018). Currently, little is known about the mechanism underlying exosomes production. Therefore, we review the molecules involved in exosomes production:

1) Rab proteins mainly modulate exosomes biogenesis through endosomes and the plasma membrane. Rab proteins determine the specificity of organelle membranes, recruit mechanical effectors, and mediate organelle dynamics. Ostrowski et al. (Ostrowski et al., 2010) found that Rab27a and Rab27b are involved in the biogenesis and localization of MVBs, in which Rab27a assembles plasma membrane microdomains by regulating plasma membrane PIP2 dynamics and participates in vesicle budding from the plasma membrane. Rab35 can be localized to the plasma membrane and regulate its PIP2 levels, which may contribute to exosomes formation (Hsu et al., 2010). Rab11 is induced by calcium and affects exosomes biogenesis (Savina et al., 2002).
2) The Ral/Arf6/PLD2/Syntenin Alix axis regulates the biogenesis of exosomes. Ral proteins regulate the ADP-ribosylation factor 6 and phospholipase D2, triggering the secretion of Syntenin and Alix to help the formation of exosomes at the membrane (Gurunathan et al., 2019).
3) The endosomal sorting complex required for transport (ESCRT) plays a pivotal role in driving both exosomal and ectosomal biogenesis (Hade et al., 2021). Hanson et al. (Hanson and Cashikar, 2012) explored about 30 different proteins organized into four machinery complexes, respectively ESCRT-0, -I, -II, and -III. The ESCRT-0 complex may help with recognizing and sorting ubiquitinated intracellular cargos to degrade lysosomes. ESCRT-I and -II are devoted to the formation of buds from membranes, thus sequestering vesicles. ESCRT-III plays a role in vesicle scission. These biogenesis events involve tetraspanins such as CD63, CD9, CD37, CD82, and CD81 (Juan and Fürthauer, 2018).
4) Autophagy is involved in exosomes secretion. Previous research demonstrated that knockout of Atg5, an autophagy-related gene expressed in cancer, decreases exosomes production. In contrast, the CRISPR/Cas9 -mediated knockout of Atg5 neurons leads to an increase in exosomes production (Pegtel and Gould, 2019).

Because of these origin, exosomes could carry various cytoplasmic and membrane components, including lipids, enzymes, transcription factors, extracellular matrix proteins, and nucleic materials, such as mtDNA, ssDNA, dsDNA, miRNA, mRNA, and ncRNA (Huang et al., 2021). Cell adhesion molecules, integrins, tetraspanins, and MHC I/II proteins are common among all exosomes. Some fusion and transferring proteins, such as Rab2, Rab7, annexins, flotillin, heat-shock proteins, cytoskeleton proteins, and the ALG2 interacting protein X, are non-specific exosomal proteins (Nikfarjam et al., 2020).

MSCderived exosomes (MSC-Exos) are being extensively exploited to develop novel regenerative strategies for numerous diseases and provide a favorable microenvironment that enhances tissue-damage repair (Miao et al., 2021). Intriguingly, their numerous advantages over MSC, such as increased viability, higher uptake, lower immune response, reduced risk of embolism, and potential to cross the blood-brain barrier, have rendered MSC-Exos a promising candidate emerging as an effective “cell-free” therapeutic approach in the field of regenerative medicine (Pant et al., 2021). For instance, Li S et al. (Li et al., 2021) used exosomes generated from dental pulp stem cells (DPSC-Exos) to treat cerebral ischaemia-reperfusion (I/R) injury, they found that DPSC-Exos remitted brain oedema, cerebral infarction and neurological repair in I/R mice, and reduced inflammatory response like IL-6, IL-1β and TNF-α. Xin et al. (Xin et al., 2013) The miR-133b in exosomes derived from MSC could be transferred to neural cells, which improved functional recovery in a stroke rat model and enhanced neurite remodeling and angiogenesis. Exosomes have therapeutic effects through miR-326 overexpression in inflammatory bowel disease, relapsing-remitting multiple sclerosis, hepatocellular carcinoma, Parkinson’s disease, and osteoarthritis (Azimi et al., 2019; Wang et al., 2020; Zhang et al., 2021a). The circRNAs are abundant in exosomes and can be transferred into other cells to regulate biological functions. CircZC3H7B is an important exosomal circRNA during chondrogenesis in MSC, and overexpression of exosomal CircZC3H7B promoted cartilage-specific gene and protein expression through the miR-3677-3p/Sox9 axis. Therefore, CircZC3H7B overexpressing exosomes may alleviate cartilage degradation, suppressing OA progression and enhancing cartilage repair (Mao et al., 2021). Exosomes exhibit a diversified repertoire of functional ncRNAs and potentially transfer these biologically active transcripts to the recipient cells, where they modulate diverse functions (Pant et al., 2021). Li et al. (Li et al., 2019) discovered that, in a rat model of myocardial infarction (MI), miR-301 of exosomes released by MSC prevented cardiac autophagy and improved myocardial function. Compared with the sham group, high expression of miR-301 in the exosome group resulted in a higher left ventricular ejection fraction and shortened left ventricular fraction, as well as a lower left ventricular end-diastolic diameter and left ventricular end-systolic diameter in rats with MI, a decreased ratio of LC3-II/LC3-I, and upregulation of p62, indicating a lower level of autophagy than the sham model. These preclinical data proved the reliability of exosome therapy and laid the foundation for its clinical application. In recent years, numerous studies have explored MSC-Exos in clinical settings. At present, there are 22 clinical trials in the recruitment and active phases, only 5 have been completed. For example, Shi et al. (Shi et al., 2021) reported that nebulized exosomes derived from human adipose-tissue-derived MSC (haMSC-Exos) exerted protective effects in pneumonia caused by *P. aeruginosa* in mice and that nebulization with haMSCs-Exos in healthy volunteers did not cause serious side effects (Trial Registration: MEXVT, NCT04313647). Zhu et al. (Zhu et al., 2022) showed that inhaling a dose of clinical grade haMSC-Exos up to a total amount of 2.0 × 109 nanovesicles for 5 consecutive days was feasible in COVID-19 patients, with no adverse events, clinical instability, or dose-relevant toxicity. CT imaging revealed the safety profile of the lungs after nebulization with haMSC-Exos (Trial Registration: MEXCOVID, NCT04276987).

Microvesicles (MVs), also called shedding vesicles, are another type of subcellular physiological vehicle of EVs present in all body fluids and cell types (Guo et al., 2019). MVs express parental cell membrane markers and contain intracellular information. They exhibit similar characteristics to exosomes, such as physical, and functional properties, but differ from exosomes in size, cargo, mechanism, and other functions because of their diverse generation types (Inal et al., 2012; Tricarico et al., 2017). Apoptotic bodies (ApoBDs), a distinct subclass of EVs, remain largely unexplored and are released by cells undergoing programmed cell death termed apoptosis. ApoBDs encapsulate various cellular components, i.e., cytosol, degraded proteins, DNA fragments, or even an intact organelle (Hauser et al., 2017). During the period of apoptosis, membrane blebbing promotes the distribution of nuclear material into ApoBDs (Santavanond et al., 2021). Breakage into ApoBDs contributes to a more-efficient clearance of apoptotic cells and is important in controlling immune responses (Xu et al., 2019). MSC lysates are derived from repeated cell freeze–thaw cycles. Its soluble factors are similar to MSC, which possess the anti-apoptotic activity and reduce tissue damage by inhibiting apoptosis and promoting regeneration (Nishikawa et al., 2021).

Translational potentials of nanotechnology, devices, and materials have become an emerging area in medicine, like the application of liposomes, hydrogels, et al., this allows high-performance and sustainable delivery of biomolecules into a specific location (Khayambashi et al., 2021; Ye and Zhang, 2022). Hydrogels encapsulated exosomes accelerating the process of therapeutic benefits of exosomes in the field of diabetes mellitus, spinal cord injury, bone tissue engineering, et al. (Wang, 2018; Khayambashi et al., 2021). Due to the special hydrophilic and cross-linking structure that hydrogels could control drug release and maintain its biological activity (Wang et al., 2017). All special properties of hydrogels mainly include following charateristics (Guan et al., 2017; Ye and Zhang, 2022): 1) The hydrophilic porous structure facilitates the absorption and retention of water while maintaining structural integrity and allowing the free diffusion of granular materials. 2) The Young’s modulus and atomic mechanics of hydrogels are beneficial to cell migration and proliferation in biomaterial scaffolds. 3) Hydrogels exhibit reversible volume changes. 4) Hydrogels are formed by cross-linked monomer-polymer networks with covalent and non-covalent bonds, which enhance their adaptability to microenvironment and can highly mimic tissue properties. 5) Hydrogels degradation can support cell growth in tissue microenvironment. All of these functional characteristics lead to versatility of hydrogels, moreover, constantly modified hydrogels could augment these strengthens in advance, which in turn to treat all kinds of diseases, improve cellular migration, proliferation, and differentiation in tissue engineering, regenerative medicine, adhesive medicine, cell-encapsulation matrices, and drug-delivery systems [37.40].

In this review article, we emphasized the current knowledge on the molecular and cellular mechanisms responsible for the characteristics of culture-condition stimulated exosomes or their loaded hydrogels in comparison with other extracellular vesicles or MSC lysates. These issues will give a better understanding for scholars to explore exosomes or other EVs. For this purpose, an extensive literature review was carried out in July 2022 across several databases from 2002 to the present.

## 2 Comparison of exosomes, microvesicles, apoptotic bodies, and MSC lysates

The advantages of exosomes, microvesicles, apoptotic bodies, and MSC lysates have attracted the attention of most scholars in this field. However, rare reports disagree in their differences. In this review, we will clarify their characteristics and functions and discuss the differences between them, to help researchers better understand and master their performance.

### 2.1 Comparison of exosomes with MVs

#### 2.1.1 Function and application of MVs in regenerative medicine

MVs, also known as ectosomes, are heterogeneous membrane-bound sacs that play an important role in cell-cell communication, tissue homeostasis, cell differentiation, and organ development and remodeling (Ratajczak and Ratajczak, 2020). The biogenesis of MVs is achieved by foaming and squeezing the cell’s plasma membrane directly outward to release the new microvesicles into the extracellular space (Shifrin et al., 2013). Membrane curvature and rigidity affect the plasma membrane protein and lipid components of membrane blebbing, such as plasma membrane phospholipid redistribution and coordination of the actomyosin contractile machinery, which render MVs a unique mechanism compared with exosomes (D'Souza-Schorey and Clancy, 2012; McMahon and Boucrot, 2015). Increased calcium ion concentrations activate calcium dependent scramblase and inhibit translocase activity, which prevents the transport of phosphatidylserine and phosphatidylethanolamine to the inner side of the membrane, thereby disrupting the phospholipid composition of the plasma membrane. Thus, MVs germinate from cell membranes containing phosphatidylserine and phosphatidylethanolamine (Suzuki et al., 2013; Hashemi Tayer et al., 2019). However, MVs are not simply produced *via* the random cell sampling of cellular components, rather, through a selection of proteins and nucleic acids for recruitment into MVs, these unique proteins expressed in the MVs membrane can define the cell target (D'Souza-Schorey and Clancy, 2012). ARF6 is a key regulator of MVs that targets specific protein cargo, such as VAMP3, β-1 integrin, and MHC-I. ARF6 phosphorylates the myosin light chain to activate the extracellular signal-regulated kinase and mediate peripheral actin remodeling, cell invasion, and endocytic trafficking (Muralidharan-Chari et al., 2009). In addition to ARF6, Rab22a reportedly co-localizes to MVs, thus selectively recruiting proteins, however, in general, this occurs under hypoxic conditions (Wang et al., 2014). Furthermore, specific v-SNAREs participate in endosomal trafficking, and the MT1-MMP cargo associated with VAMP3 could shed MVs (Clancy et al., 2015). CSE1L is a functional nuclear export protein in melanoma MVs (Liao et al., 2012). Rho is a GTPase-family protein that activates the Rho-associated kinase, which enhances myosin phosphorylation, actin-myosin sliding, detachment of the plasma membrane from the cytoskeleton, and the release of MVs (Sedgwick et al., 2015). MVs derived from MSC have been exploited to activate regenerative programs in injured recipient cells and exert anti-inflammatory effects *via* the suppression of the Akt and STAT3 signaling pathways and *via* the inhibition T-lymphocyte functions, as well as the modification of cytokine production in dendritic cells, naive and effector T cells, and NK cells (Tricarico et al., 2017). MSC-derived MVs also show immunomodulatory effects in inflammatory or autoimmune disorders, such as uveoretinitis (Shigemoto-Kuroda et al., 2017), graft-versus-host disease (Wang et al., 2016), and type Ⅰ diabetes (Nojehdehi et al., 2018). Cesarean delivery is a growing worldwide maternal delivery technique to reduce scarring and optimize recovery, Pekarev et al. (Pekarev et al., 2021) developed a pilot study that evaluated the safety profile of MVs from mesenchymal stromal placental cells. This trial found that the intra-surgery injection of MSC-MVs was well tolerated and associated with a minimum rate of infectious post-partum complications after cesarean delivery. Moreover, Liang Z et al. (Liang et al., 2020) found that MVs modified PEI could improve transfection efficiency and gene expression levels, lead to earlier and higher levels of hBMP2 protein expression, increase ALP activity, and enhance calcium deposition at earlier time points. Subcutaneously, it was confirmed that a DBM/MVsPEI/phBMP2 scaffold not only promoted blood vessel formation but also induced bone formation. More importantly, MVs could regulate the pathogenesis and progression of atherosclerosis, stroke, coronary heart disease, and diabetes mellitus. This effect was closely correlated with the parental cells carried by MVs.

#### 2.1.2 Diversity of exosomes and MVs

Although exosomes and MVs have some specific markers, such as the heat-shock protein 70 family and tetraspanins (CD9, CD63, and CD81), they differ in size, lipid composition, content, and cellular origin (Lee et al., 2012). Both exosomes and MVs are involved in many biological processes, such as the immune response, angiogenesis, aging, and apoptosis (Ståhl et al., 2019). However, no studies have addressed the differences between these two EVs. The origins of these vesicles are diverse, which determines their differences regarding features and functions. It is essential to elucidate their compositions, including proteins, metabolites, etc. to study the diversity of these two EV subpopulations. To investigate these differences, Guan F. et al. (Guan et al., 2021) identified 112 different proteins and 50 different metabolites between exosomes and microvesicles from human plasma. Moreover, Comelli L. et al. (Comelli et al., 2014) exploited an untargeted, proteomics approach to characterize the vesicles released from vascular smooth muscle cells (VSMCs) and a preliminary protein profile of exosomes and MVs. In MVs, the most abundant classes of proteins were cytoplasmic or organelle-associated proteins and housekeeping and metabolic factors. This is not surprising because of their unselective, budding mechanism of formation. Conversely, exosomes from different phenotypes revealed a sharper peculiarity. Thus, as suggested by the high percentage of ECM and ECM related proteins and CAMs, they seem to play an important role in outward or cell-to-cell signaling. No mitochondrial proteins were identified in exosomes, whereas nuclear proteins were more numerous in exosomes than microvesicles. The comparison between exosomes and MVs from quiescent and activated VSMCs evidenced 29 differentially expressed proteins. MVs carried several proteins involved in vesicle trafficking, whereas exosomes had focal adhesion and ECM-related factors. Although preliminary, these data are promising for identifying potential circulating markers of a cell state.

Conceivably, the properties of exosomes and MVs could generate different environmental signals. MVs sprouting from the cell membrane appears to primarily reflect the transient aggregation of elements around the plasma membrane of cells, including cell cortical content, which is located directly beneath the cell membrane, and membrane-associated proteins involved in vesicle formation and transport (Camussi, 2022). Conversely, exosomes are produced by more-specific mechanisms that appear to take up specific panels of proteins, the combination of which depends on the phenotypic state of the cell (Fu and Wu, 2021).

### 2.2 Comparison of exosomes with ApoBDs

#### 2.2.1 Function and application of ApoBDs

ApoBDs are characteristic membrane blebs that are generated by cell fragmentation when the cytoskeleton breaks at the beginning of apoptosis (Crescitelli et al., 2013). ApoBDs are produced during the disintegration of apoptotic cells. The nucleus and cytoplasm rapidly wrap into multiple tightly membrane-bound vesicles of different sizes (Blander, 2017). Cell components in cytoplasmic processes randomly form apoBDs; therefore, some ApoBDs consist almost entirely of condensed nuclear chromatin, whereas others carry cytoplasmic components (Akers et al., 2013). For these reasons, ApoBDs are subdivided into two groups: nuclear apoptotic bodies and cytoplasmic apoptotic bodies (Hauser et al., 2017). Recent efforts revealed that ApoBDs are key messengers in regulating cell clearance, tissue homeostasis, pathogen dissemination, and immunity, thus implementing their therapeutic potential (Atkin-Smith et al., 2019; Xu et al., 2019). First, ApoBDs could rapidly remove cell debris by mediating macrophages or immature dendritic cells, for instance, billions of cells undergo apoptosis every day during the natural development of organisms. However, apoptotic cells are rarely observed under these physiological conditions, suggesting their rapid clearance in homeostasis, in which ApoBDs play a crucial role (Poon et al., 2014; Nagata, 2018; Brock et al., 2019). Second, ApoBDs could be used as vaccines and immunotherapies because they reportedly contain pathogen-derived antigens when they stem from tumor conditions and facilitate the response of adaptive T cells during cross-antigen presentation (Hauser et al., 2017). For example, in two subsequent independent vaccination studies, autologous tumor ApoBD-pulsed dendritic cells appeared to trigger a leukemia-specific CD8^+^ T-cell response, T-cell–tumor cell aggregation, and reduced regulatory T-cell levels in 40–60% of the patient cohorts, without dose-limiting toxicity and autoimmunity, however, clinical trials of these effects are lacking, which warrants further studies (Palma et al., 2012). Third, ApoBDs released by stem cells or differentiated cells could also be used in tissue regeneration. For example, Liu J. et al. (Liu et al., 2020a) showed that ApoBDs derived from BMSCs promoted cutaneous wound healing by triggering the polarization of macrophages toward the M2 phenotype. In addition, the functional converted macrophages further enhanced fibroblasts’ migration and proliferation abilities, which facilitated the wound-healing process. Similarly, mature-osteoclast-derived ApoBDs also promoted preosteogenic cell viability and differentiation, which is reported to promote survival and osteoblast differentiation through the NF-κB receptor activator mediated PI3K/Akt/mTOR/protein S6 kinase signaling pathway (Ma et al., 2019). Using a zebrafish model, Brock et al. (Brock et al., 2019) found that basic stem cell-derived ApoBDs could induce division in adjacent stem cells through the Wnt8a signaling pathway. This local apoptosis-induced proliferation could maintain cell number and homeostasis. Fourth, according to the current reference, ApoBDs transfer different molecules (DNA, miRNA, and proteins), effectively regulate various aspects of the phagocytic/recipient cells and protect vascular and cell migration, which provides evidence that ApoBDs are potential drug-delivery systems (Liu et al., 2020b). Finally, ApoBDs could serve as diagnostic tools, such as in the case of graft-versus-host disease, in which ApoBDs were abundant in bodily fluids. The recently developed flow cytometry based approach would analyze these disease-associated ApoBDs quickly, thus providing evidence that ApoBDs could be a rapid, accurate, and minimally invasive diagnostic tool (Jiang et al., 2016; Atkin-Smith et al., 2017).

#### 2.2.2 Diversity of exosomes and ApoBDs

Unlike the exosomes generated by normal viable cells, ApoBDs are a distinct type of EVs that undergo programmed cell death (Crescitelli et al., 2013). ApoBDs have a larger size and carry proteins, lipids, RNA, and DNA, moreover, they significantly affect their downstream cells or recipient cells (Xu et al., 2019). For example, the osteoclast derived ApoBDs have a higher RANK level and osteogenic potency compared with exosomes, moreover, ApoBDs in regenerative therapy, especially organ transplantation, could offer a better safety advantage, as they did not induce the inflammatory response and graft rejection, as observed for other EVs (Ma et al., 2019). Clearance defects in the formation of ApoBDs may contribute to the development of autoimmunity in autoimmune diseases. Some studies have shown that ApoBDs have a stronger procoagulant effect on cancer cells. These results highlight the role of ApoBDs in promoting thrombogenesis and anticancer immunity (Xu et al., 2019). Islet inflammation caused by beta cell failure and apoptosis is associated with autoimmune type Ⅰ diabetes. Giri KR et al. (Giri et al., 2020) performed comparisons between ApoBDs, MVs, and exosomes isolated from MIN6 beta cells and found 5-, 2-, and 4-fold increases in the number of particles observed for ApoBDs, MVs, and exosomes, respectively, under inflammatory conditions. Conceivably, ApoBDs from MIN6 beta cells contains a source of chemoattractants, beta self-antigens, and danger signals that could interfere with the otherwise immune-silent elimination of dead cells by efferocytosis. In contrast, exosomes and MVs have a better ideal size for diffusion to secondary lymphoid organs, such as the spleen and draining lymph nodes.

### 2.3 Comparison between exosomes and MSC lysates

#### 2.3.1 Function and application of MSC lysates

Unlike cell-free MSC-conditioned medium and MSC-Exos, MSC lysates contain MSC cell-surface proteins, which might amplify their beneficial effects (Nishikawa et al., 2021). For the processing of MSC lysates, deionized H_2_O replaced culture media in each dish, followed by incubation at room temperature for 30 min and processing through three freeze–thaw cycles to dissociate lysed cell sediments. After centrifugation at 1,000 × g, MSC lysates were prepared, and the components of MSC lysates were identified (such as FGFs, TGF-β, VEGFs, and many other cytokines participating in the regenerative phase of inflammation). They may indifferently stimulate the proliferation of both normal cells and mutated cells, which are naturally selected for their high proliferation potential. They possess anti-apoptotic activity, which reduces tissue damage by inhibiting apoptosis and promotes regeneration (Albersen et al., 2010; Khubutiya et al., 2015; Hsu et al., 2018). Furthermore, MSC lysates expose soluble factors to tissues without allowing live cells to act directly on host tissues, which amplifies their positive effects. Regarding their applications, inflammatory bowel diseases (IBDs) are chronic, persistent, and intractable disorders for which no fully established therapeutic systems exist. Nishikawa T. et al. (Nishikawa et al., 2021) found that filtrated murine adipose tissue-derived MSC lysates (FADSTL) contain extract and cell-surface proteins, which have a similar effect to that of exosomes in IBD, such as anti-inflammatory action and apoptosis suppression. In FADSTL treated mice, the intestinal epithelial structure was preserved, and neutrophil infiltration was reduced, moreover, no obvious side effects were observed. Erectile dysfunction remains a major complication after radical prostatectomy. Albersen M. et al. (Albersen et al., 2010) found that nNOS content was preserved both in adipose tissue derived stem cells (ADSCs) and lysates groups, and less fibrosis and significant preservation of smooth muscle were observed in cavernous nerve (CN) crush injury. In addition, ADSC-derived lysates afford neuronal preservation and cytoprotection by inhibiting apoptosis. Acute liver failure is caused by viral hepatitis and toxic liver injury and has a high mortality rate of 80–90%. Khubutiya et al. (Khubutiya et al., 2015) found that the microRNA-like MATK, MRE11A, CHECK2, MYH11, VASP, and CDK2 are linked to cell proliferation and the inhibition of apoptosis in MSCs lysates, with these results indicating that bone marrow MSC lysates reduce necroses and increase mitotically active cells during the first hour after transplantation. Intriguingly, one study found improved glucose tolerance in high-fat-diet–fed mice, which suggests the possibility of using MSC lysates for glucose metabolism as an encouraging approach to human anti-aging intervention, however, weight loss and a decrease in lipid levels in later life can be life-threatening, therefore, Hsu MF et al. (Hsu et al., 2018) carried out a 3-year lifelong experiment. They prepared 92 rats that were randomly divided into the vehicle group and adipose-derived MSC lysates injection group and measured lifespan, spontaneous motor activity, and body composition after the whole trial. They found that the use of MSC lysates did not prolong the life span and vitality of rats, and weight loss caused by MSC lysates discord with bone and bone-free lean tissues led to imbalance of tissue growth and size maintenance in middle age, which suggests that a robust multicellular system is of particular importance.

#### 2.3.2 Diversity of exosomes and MSC lysates

Exosomes are membrane-wrapped vesicles that are mainly secreted by cells. Moreover, they are vehicles for bioinformatical communication and the exchange of genetic information between cells and can play a role in crossing biological barriers (Tang et al., 2021). Conversely, MSC lysates are the product of cell lysis that carry cell contents and cell membrane surface proteins and act directly on the damaged tissue, no obvious side effects were reported such as anaphylactic reaction that can lead to death immediately after administration (Nishikawa et al., 2021). Because they are relatively novel, there is little information on the strengths and weaknesses of exosomes and cell lysates. Fortunately, an article compared the function of human platelet lysates and their EVs, which would help understand the differences between EVs and lysates. The data established that EVs isolated from human platelet lysates are not toxic to undifferentiated neuroblastoma cells and promote the proliferation and repair of neuronal cells in a wound scratch assay. In addition, EVs isolated from human platelet lysates are not toxic regarding the formation of primary cortical neurons. However, additional research is needed to understand the application of exosomes and cell lysates in regenerative medicine and tissue repair and their specific contributions to neuroprotection and neurological recovery (Nyam-Erdene et al., 2021).

## 3 Characteristics of exosomes loaded hydrogels or under different culture conditions

Many studies have raised the possibility of using exosomes as an alternative to cell-based therapy because they retain the characteristics of the cell of origin (Gurunathan et al., 2019). Several exogenous factors affect stem cell growth, proliferation, and differentiation, such as hydrogels coating, hypoxia, 3D culture, oxidative stress (OS), osmosis, temperature, humidity, gas diffusion exchange, acidity, the rigidity of growth surfaces, cell density, and the modes of multicellular associations, which affect the secretion of exosomes (Rahimi et al., 2021). The therapeutic effect of exosomes is influenced by the diverse molecular components (e.g., protein and RNA cargoes) loaded onto them (Shafiei et al., 2021). Therefore, understanding the characteristics of exosomes under different culture conditions is of great importance.

### 3.1 Characteristics of exosomes loaded hydrogels

Exosomes are routinely administered by intravenous, subcutaneous, or intraperitoneal injections (Safari et al., 2022). However, these way greatly limit the therapeutic function because of rapid clearance and short half-life *in vivo*. In addition, choronic disease like osteoarthritis require a long recovery time (Zhang et al., 2018). Thus, how to maintain exosomes bioactivity is of extremely significance, hydrogels with interconnected networks and small pores are good options. Hydrogels are chemical cross-links between chains of hydrophilic polymers with three-dimensional structures formed by physical or chemical substances, and its rheological properties similar to extracellular matrix, which could mimic its various functions (Luo et al., 2018). Also, they have adjustable physical and mechanical properties such as elasticity and ability to retain shape, like, when injecting hydrogels, it could be a form of liquid, once arrived at wound area, it will fit perfectly to the wound site to form as solid biomaterial to play a role (Slaughter et al., 2009; Albashari et al., 2020). Study showed that exosomes in hydrogels could preserve and even increase the stability of proteins and microRNA in exosomes. Moreover, through the gradual degradation of hydrogel internetwork, exosomes are released and diffuse over the long term, which minimizes drawback caused by rapid administration of exosomes (Zhang et al., 2018). Therefore, in other words, the therapeutic effect of exosomes largely depends on the design and function of hydrogels. Yang S et al. (Yang et al., 2020a) used a sensitive injectable alginate hydrogels (ALG) and hyaluronic acid (HA) hydrogels with self-healing ability to carry exosomes derived from umbilical cord mesenchymal stem cells (HUMSC) in humans, they found that this kind of composite biological material could improve the osteogenic effect of rat calvarial bone defect model. In addition, the combination of hydrogels and exosomes could promote the healing of damaged bone, BMP2 deposition, deposition and maturation of bone collagen, and angiogenesis was increased in SD rats. Electroconductive hydrogels can accelerate spinal cord injury (SCI) repair as they match electrical and mechanical properties of neural tissue. However, electroconductive hydrogels solely implant may increase inflammation and prevent its repair efficacy. Fan L et al. (Fan et al., 2022) found that neural tissue-like electroconductive hydrogels loaded with BMSC-exosomes could be developed to treat SCI, they could regulate M2 polarization through NF-ĸB pathway, and increase axon outgrowth *via* the PTEN/PI3K/AKT/mTOR pathway, which exhibited a significant functional recovery at the early stage in an SCI mouse model. Delayed wound healing happened in diabetes mellitus (DM) patients is one of the most challenging complications, as it poses a greater risk of gangrene, amputation and even death, there are approximately 422 million registered diabetics in 2014, the number is expected to 592 million by 2035 globally. Shi Q et al. (Shi et al., 2017) found exosomes derived from gingival mesenchymal stem cells (GMSCs) loading with chitosan/silk hydrogel sponge effectively promoted healing of diabetic skin defects, histological analysis clearly revealed more neo-epithelium and collagen, and the highest microvessel density and nerve density in this assembled group. In another study, corneal diseases have caused to approximately 12 million people worldwide blinded. Corneal injury forms scar tissue, which is manifested as permanent corneal opacity. It is one of the most serious diseases causing blindness due to the low ability of corneal regeneration. In order to treat this disease, Tang Q et al. (Tang et al., 2022) used a thermosensitive chitosan-based hydrogels (CHI hydrogel) sustained-release iPSC-MSC exosomes, after a series of study, they found that this iPSC-MSC exosomes loaded CHI hydrogel could effectively promote the repair of damaged corneal epithelium and stromal layer, downregulating mRNA expression of collagen type Iα-1, type Vα-1 and type Vα-2 in corneal stroma and lessening scar formation *in vivo*. Furthermore, iPSC-MSCs exosomes contain miR-432-5p, it could restrain translocation-associated membrane protein 2, which is an important regulator of collagen biosynthesis in corneal stromal stem cells, and could inhibit the deposition of extracellular matrix. Their findings indicated that thermosensitive CHI hydrogel based on iPSC-MSCs exosomes is a promising clinical treatment technique for various corneal diseases. Desktop-stereolithography (SLA) 3D printing could be a more promising strategy for regenerative engineering. A recent study successfully fabricated a 3D-printed ECM/GelMA/exosomes scaffold with SLA technology in osteochondral defects, this scaffold promoted chondrocyte migration in the defect regions and the sustainable release of exosomes, which could be internalized by chondrocytes (Chen et al., 2019). Moreover, in OA, chondrocytes would lose metabolic flexibility, reduce cellular mitochondrial biogenesis, and augment mitochondrial DNA, reducing ATP synthesis. Furthermore, an ECM/GelMA/exosome scaffold enhanced dysfunctional mitochondria in chondrocytes by supplementing mitochondria-related proteins in exosomes and polarizing the synovial macrophage response toward the M2 phenotype (Chen et al., 2019).

### 3.2 Exosomes under hypoxia culture

Oxygen concentration is essential for the proliferation, differentiation, and self-renewal of MSC. MSC are generally exposed to an oxygen concentration of 21% *in vitro* (Hu et al., 2014). Many MSC usually exist in low oxygen (2–8% O_2_ or even lower) in organisms (Mohyeldin et al., 2010). A study found that the exosomes released from MSC cultured using a condition similar to peripheral arterial disease (1% O_2_) included various pro-angiogenic factors (Anderson et al., 2016). Reduced oxygen tension in tissues activates the hypoxia-inducible factor 1α, which induces the transcription of angiogenic genes, such as the vascular endothelial growth factor and MSC chemoattractant stromal cell-derived factor 1 genes (Ceradini et al., 2004; Berniakovich and Giorgio, 2013). Hypoxic preconditioning of MSC can enhance their paracrine effects (Madrigal et al., 2014). Bone fracture repair is a complex process, among many studies, angiogenesis has been recognized to play an important role in bone metabolism. Exosomes from MSC grown in hypoxic conditions include abundant pro-angiogenic factors, which have therapeutic implications (Zhu et al., 2018). Liu W. et al. (Liu et al., 2020c) reported a mechanism *via* which Hypo-Exos could accelerate the healing of bone fractures by activating HIF-1α to transfer miR-126 to endothelial cells *via* the SPRED1/Ras/Erk pathway. In diabetes diseases, hypoxia can always decrease beta cell viability, transplanted islets usually suffer hypoxia because of poor vascularity and low oxygen tension, which seriously interferes with the quality of life of these patients. Recently, researchers revealed that Hypo-MSC-Exos are significant mediators that promote angiogenesis and improve the survival of beta cells against hypoxia-induced apoptosis by inhibiting p38 MAPK phosphorylation, which improved encapsulated islet survival and benefited patients with diabetes to a great extent (Chen et al., 2020). Ischemic heart disease is the leading cause of mortality worldwide, and cardiosphere-derived cells (CDCs) reverse post-MI injury. However, exosomes secreted by CDCs under hypoxic conditions enhanced tube formation to a greater extent and upregulated miR-210, miR-130a, and miR-126, which are pro-angiogenic miRNAs present in exosomes. This study suggests the benefit of hypoxic CDCs exosomes for treating cardiac diseases (Namazi et al., 2018a). Another study also verified the anti-apoptotic effect of CDCs-derived exosomes in cardiomyocyte protection against cocultured CDCs with CoCl_2_-induced hypoxia (Namazi et al., 2018b). Acute myocardial infarction (AMI) is another type of cardiovascular disease. Cardiac fibrosis can occur secondary to AMI, and previous research has reported that exosomes from endothelial colony-forming cells (ECFCs) exposed to hypoxia greatly increased cardiac fibroblast activation, whereas miR-10b-5p-enriched exosomes extracted from ECFCs activated the TGF-β signaling pathway to reduce fibroblast activation after AMI (Namazi et al., 2018b).

Exosomes released from different sources have different functions. Hypoxia is a pivotal feature in all types of solid tumors that contributes to tumor development, drug resistance, and distant metastasis. It triggers tumor cells to produce exosomes, which enhance cancer cell survival, promote the metastatic niche, affect various immune cells by hypoxia-induced immunosuppression, and promote cancer progression (Berchem et al., 2015; Kore et al., 2018; Kumar and Deep, 2020). For example, RNAs are reported to be the predominant molecules in tumor-cell-derived exosomes, such as miRNAs, cirRNAs, and lncRNAs (Conigliaro et al., 2015). Urothelial cancer-associated 1 (UCA1) was highly expressed in bladder cancer and regulated the cAMP response element-binding protein, chromatin remodeling factor, phosphoinositide 3-kinase, protein kinase B, and Wnt pathways, to promote bladder cancer cell proliferation (Wang et al., 2006; Wang et al., 2008; Fan et al., 2014). Xue M. et al. (Xue et al., 2017) found that exosomes derived from bladder cancer cells under a hostile hypoxic microenvironment and carrying lncRNA-UCA1 promoted tumor progression, moreover, its exosomal lncRNA-UCA1 in human serum may provide a potent diagnostic biomarker for bladder cancer. Nevertheless, the characteristics of exosomes from immune cells under a hypoxia environment have a positive effect. One study reported that exosomes from natural killer (NK) cells, regarded as the body’s first line of defense against viral and bacterial infections, increased under hypoxic conditions and enhanced cytotoxicity and apoptosis. More interestingly, exosomes derived from NK cells under hypoxic conditions are more potent than those stemming from normal NK cells in inhibiting the proliferation and migration of cancer cells and promoting their apoptosis (Jiang et al., 2021).

### 3.3 Exosomes under three-dimensional (3D) culture

Recently, three-dimensional scaffolds have attracted much attention in the field of regenerative medicine. Here, we introduce a 3D scaffold containing natural materials, synthetic materials, and 3D bioprinting in exosome-based regenerative therapy (Chang et al., 2020). Biomaterials prepared with exosomes elicited a therapeutic effect in injured tissues with no disadvantages because of their direct fusion with target cells, easy usage, and absence of immunological rejection and tumorigenesis (Sun et al., 2019). Treatment with exosomes requires a large amount of MSC-Exos. According to statistics, one mouse or rat needs approximately 20–200 μg of exosomes. One patient in clinical testing requires 100 μg of exosomes/kg of body weight (Nassar et al., 2016; Shen et al., 2016; Kamerkar et al., 2017), their low output limits their large-scale research and applications (He et al., 2018). Therefore, studies have reported the use of a hollow fiber bioreactor-based 3D culture system to culture husMSC, which resulted in a higher production yield (7.5-fold) and purity (6.5-fold) of exosomes compared with 2D culture (Yan and Wu, 2020). Moreover, when they are produced from tangential flow filtration culture, the yield of exosomes is 7-fold that of ordinary 3D culture (Haraszti et al., 2018). Furthermore, 3D-Exos has therapeutic efficacy in various diseases. For example, husMSC-Exos from a hollow fiber bioreactor-based 3D culture system could alleviate cisplatin-induced acute kidney injury by attenuating the pathological changes in renal tubules, reducing inflammatory factors, and repressing T-cell and macrophage infiltration, to an extent greater than that observed for 2D-Exos (Cao et al., 2020). Another study also found that exosomes produced from 3D cultures of husMSC in a hollow fiber bioreactor could stimulate cell proliferation, migration, and matrix synthesis and inhibit apoptosis in cartilage defects (Yan and Wu, 2020). In turn, there is no effective pharmacological approach to cure traumatic brain injury (TBI). Therefore, a combination of exosomes and 3D collagen scaffolds was designed to solve this problem. It has been verified that collagen scaffolds mimic the natural extracellular matrix, and MSC-Exos encapsulated in collagen scaffolds promote neurovascular remodeling, suppress the expression of axonal growth inhibitory molecules (such as neurocan and nogo-A), and reduce neuroinflammation, which suggests that exosomes in 3D collagen scaffolds may represent a novel and safer therapeutic cell-free treatment for TBI (Zhang et al., 2017). Hydroxyapatite scaffolds are artificial devices that are broadly used in tissue regeneration. Previously, BMSCs cultured on hydroxyapatite scaffolds to produce 3D exosomes enhanced angiogenesis by secreting increased levels of HMGB1, VEGF, and CD31, thus activating the PI3K/AKT axis (Gao et al., 2020). Alzheimer’s disease (AD) is the most common type of dementia; unfortunately, no effective treatments prevent neuronal cell death, Aβ deposition, and cognitive decline in patients with AD. Recent research revealed that exosomes derived from hUMSCs in porous scaffolds could cut down Aβ generation by increasing the expression of ADAM10 and downregulating BACE1, NEP, IDE, and HSP70; moreover, they attenuated inflammation and OS, inhibited microglial activity, and improved spatial learning and memory functions, thus providing insights into a therapeutic intervention for AD (Yang et al., 2020b).

### 3.4 Exosomes under oxidative stress

Aerobic metabolism is linked to reactive oxygen species (ROS) and reactive nitrogen species (RNS), which are derivatives of oxygen and nitrogen, respectively, that play an important role in physiological and pathological processes (Moldogazieva et al., 2018). Excessive ROS and RNS may induce dangerous consequences for cell integrity and function. In contrast, aerobic cells counteract the harmful effects of ROS by producing antioxidant defenses, including thioredoxin, peroxiredoxin, glutathione peroxidase, etc. (Angelova and Abramov, 2016; Zhang et al., 2021b). The antioxidant machinery modulates the redox status of cells to maintain the aerobic metabolism balance. However, once the ROS levels exceed the antioxidant defenses, OS occurs, a harmful condition that potentiates various pathologies, such as cancer, diabetes, AD, and parkinsonism (Sies et al., 2017; Butterfield and Boyd-Kimball, 2020). OS could also affect the exosomes released by all cells, with oxidative-stress-related exosomes exerting both beneficial and harmful effects depending on their cargo (ROS-related enzymes or oxidized molecules, respectively) (Chiaradia et al., 2021).

The first issue to consider in this context is the increase in exosomes release under OS. Numerous studies have demonstrated that different pro-oxidant stimuli increase the release of exosomes. For instance, the retinal pigment epithelium (RPE) is a single-cell layer continuously exposed to OS, leading to neovascularization and blinding diseases, Atienzar-Aroca et al. (Atienzar-Aroca et al., 2016) found that ARPE-19 cells treated with ethanol secreted many exosomes and expressed membrane-bound VEGFR-1 and VEGFR-2, which were not found in other cases, these extra cargoes promoted vasculogenesis/angiogenesis. Takasugi et al. (Takasugi et al., 2017) showed that the exposure of the human retinal pigment-containing epithelial RPE-1 cells to the ROS inducer doxorubicin also triggered the release of many exosomes compared with control cells. Furthermore, exosomes under OS have protective signals depending on their specific biochemical cargo (Chiaradia et al., 2021). For example, exosomes released by mouse mast cell lines exposed to H_2_O_2_ induced tolerance to OS in target cells, enhancing the viability of cells exposed to H_2_O_2_ compared with exosomes released under normal conditions, this protective effect was mediated by exosomal mRNA, as assessed using microarray analysis (Eldh et al., 2010). Saeed-Zidane et al. (Saeed-Zidane et al., 2017) found that OS could lead to abnormal growth, function, and apoptosis in granulosa cells during ovarian follicle growth and oocyte maturation, however, its released exosomes could carry antitoxidants, when exosomes released by granulosa cells under OS were cocultured with normal granulosa cells, normal granulosa cells were enriched with the mRNA of Nrf2 and the antioxidants CAT, PRDX1, and TXN1, thus, ROS accumulation was reduced, and the G_0_/G_1_ phase was increased, the G_2_/M phase was decreased, which exhibited a proliferation rate, these results suggest that oxidative stress-released exosomes carry antioxidant molecules against the OS condition. Another study showed that exosomes secreted from oxidative-stress-activated endothelial cells (ECs) could stimulate angiogenesis by inhibiting miR-92a-3p expression in recipient ECs (Li et al., 2020). Conversely, exosomes under OS also had harmful signals in target cells because of their special cargo (Chiaradia et al., 2021). Exosomes play a crucial role in feto-maternal communication and pregnancy. During pregnancy, amnion epithelial cell (AEC)-derived exosomes are increased and participate in feto-maternal crosstalk. However, exosomes from AECs carry a unique cargo under OS and cause inflammation in fetal and maternal tissues by mediating signaling cascades such as adhesion molecules, Ras-related protein, p53 signaling, MAPK signaling, and TNF signaling (Shahin et al., 2021). Exosomes from macrophages derived from bone marrow can transfer NOX2 to injured axons, thus eliciting an increase in ROS levels (Hervera et al., 2018).

## 4 Future perspectives and concluding remarks

**FIGURE 1:**
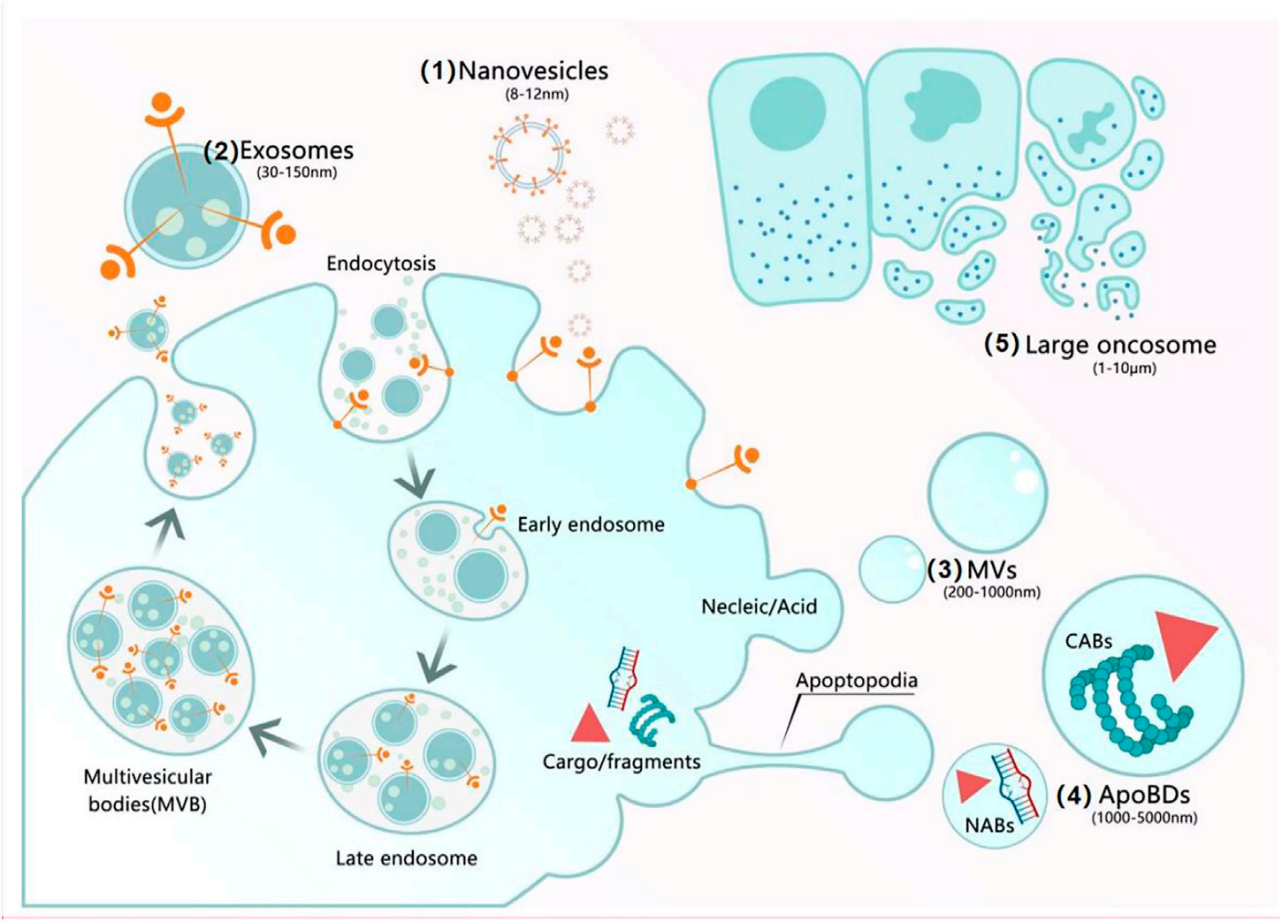
Mechanisms of secretion of EVs. (1) Nanovesicles (8–12 nm) have an unknown origin. (2) Exosomes (30–150 nm): After most endosomes mature to multivesicular bodies (MVBs) or late endosomes, their contents include RNAs, proteins, and lipids, the fusion of MVBs with the cell membrane triggers the release of ILVs as exosomes. (3) MVs (200–1,000 nm) result from the formation of buds containing cytoplasmic proteins, nucleic acids, and membrane proteins. (4) ApoBDs (1,000–5,000 nm) are generated by cell fragmentation when the cytoskeleton breaks at the beginning of apoptosis and can be subdivided into two groups: nuclear apoptotic bodies and cytoplasmic apoptotic bodies. (5) Large oncosomes (1–10 μm) result from budding from the plasma membrane.

**FIGURE 2:**
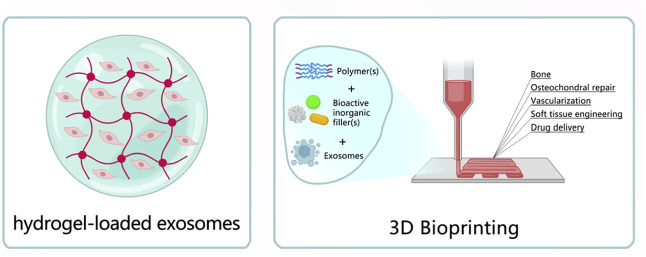
Exosomes loaded hydrogels. (1) Hydrogels carried exosomes. (2) 3D bioprinting.

**FIGURE 3:**
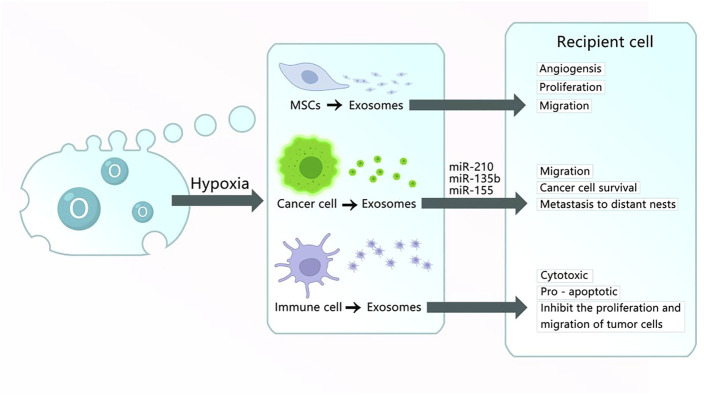
Exosomes obtained under hypoxic culture. (1) Exosomes derived from MSC cultured in hypoxic conditions carry HIF-1α, VEGF-A, EGFR, and other materials into the recipient cell to promote the angiogenesis, proliferation, and migration of damaged tissues. (2) Exosomes derived from cancer cells cultured in hypoxic conditions carry miR-210, miR-135b, miR-155, and other RNA or proteins into recipient cells to promote angiogenesis, migration, cancer cell survival, and metastasis to distant nests. (3) Exosomes derived from immune cells cultured in hypoxic conditions have cytotoxic and pro-apoptotic effects and inhibit the proliferation and migration of tumor cells.

**FIGURE 4:**
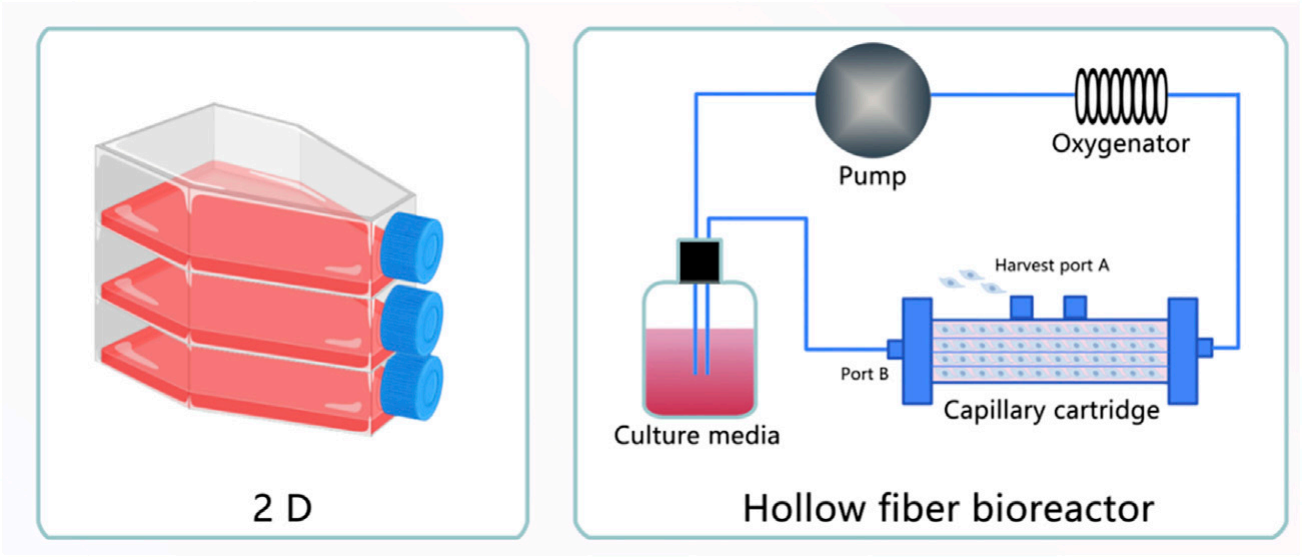
Exosomes obtained under three-dimensional (3D) Culture. (1) Exosomes extracted from MSC cultured in five-layer flasks. (2) Exosomes extracted from MSC cultured in a hollow fiber bioreactor.

**FIGURE 5:**
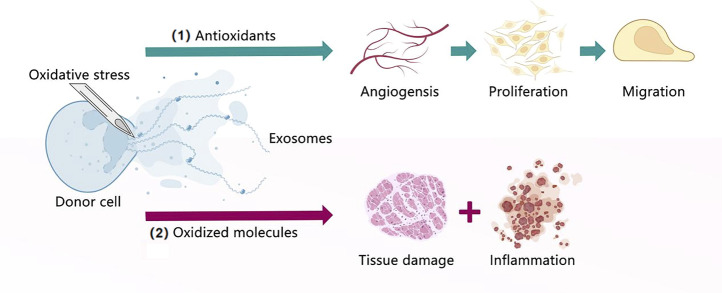
Exosomes under oxidative stress. (1) Exosomes extracted from donor cells cultured under oxidative stress contain antioxidants that can promote angiogenesis, proliferation, and migration. (2) Exosomes extracted from donor cells cultured under oxidative stress contain oxidized molecules that lead to tissue damage and inflammation.

Exosomes-based therapeutic approaches are widely used worldwide, with better therapeutic effects compared with MSC themselves. They can cross biological barriers, be modified to load molecular drugs, have few side effects, are relatively non-immunogenic, and remain active during storage. Moreover, they are essential for tissue damage repair, inflammation suppression, and immune system regulation. They contain DNA, RNA, and proteins, which alter their operational status from mother cells to targeted cells. As described above, regarding their comparison with other EVs and MSC lysates, it is clear that exosomes are mainly produced by endocytosis, whereas MVs are principally produced by budding. Therefore, exosomes contain extracellular matrix proteins, whereas MVs mainly contain membrane proteins. Furthermore, ApoBDs are mainly the products of programmed cell death, and their sizes vary greatly. The scavenging ability of ApoBDs endows them with an improved ability to regulate autoimmunity compared with other vesicles. MSC lysates are the product of cell lysis and contain cell-surface proteins and dissolved products in the cytoplasm, moreover, they have no immune rejection compared with cells, and their role in the field of regeneration is similar to that of exosomes and cell supernatants. However, there are no reports comparing exosomes with lysates. Currently, many studies are being conducted to explore the releasing ability and function of exosomes through the culture of cells under different conditions. Exosomes in hydrogels increase the stability of proteins and microRNA in exosomes, and its gradual degradation and release minimize the defects caused by rapid administration of exosomes, exosomes derived from hypoxic MSC promoted cell proliferation and tissue repair, reduced apoptosis, and induced angiogenesis. In contrast, exosomes derived from hypoxic tumor cells improved the survival rate of cancer cells, triggered distant metastasis of cancer nests, and led to drug resistance. In turn, exosomes derived from hypoxic immune cells promoted apoptosis and inhibited the proliferation and migration of tumor cells. Moreover, exosomes obtained under three-dimensional (3D) culture exhibited enhanced unleashing from cells, increased output, and a heightened damage-repair ability. Finally, exosomes derived from cells undergoing OS exhibited increased production and generated antioxidant factors to resist OS (Figures 1–5) (Tables 1–3).

**TABLE 1:** Clinical studies of mesenchymal stem cell-derived exosomes that were completed up to August 2022 (https://clinicaltrials.gov).

NCT.No	Item	Responsible party	Treatment of disease	Related DOI
NCT04313647	A Tolerance Clinical Study on Aerosol Inhalation of Mesenchymal Stem Cells Exosomes In Healthy Volunteers	Ruijin Hospital	Aerosol inhalation treatment	10.1002/jev2.12134
NCT04276987	A Pilot Clinical Study on Inhalation of Mesenchymal Stem Cells Exosomes Treating Severe Novel Coronavirus Pneumonia	Ruijin Hospital	novel coronavirus pneumonia	10.1186/s13287-022-02900-5
NCT03562715	Role of Mesenchymal Stem Cells Exosomes Derived microRNAs; miR-136, miR-494 and miR-495 in Pre-eclampsia Diagnosis and Evaluation	Nadine wagdi maurice, Cairo University	Preeclampsia	10.1016/j.abb.2018.09.023
NCT04493242	Extracellular Vesicle Infusion Treatment for COVID-19 Associated ARDS (EXIT-COVID19)	Vikram Sengupta, Direct Biologics, LLC	COVID-19 Associated ARDS	10.1089/scd.2020.0080
NCT04491240	Evaluation of Safety and Efficiency of Method of Exosome Inhalation in SARS-CoV-2 Associated Pneumonia. (COVID-19EXO)	State-Financed Health Facility “Samara Regional Medical Center Dinasty"	SARS-CoV-2 Associated Pneumonia	no published

**TABLE 2:** The list of sectional diverse exosomes and hydrogels treatment to diseases.

Source of exosomes	hydrogel material	Treatment	References
Umbilical cord mesenchymal stem cells	ALG and HA hydrogels	Calvarial bone defect	Yang et al. (2020a)
Bone marrow mesenchymal stem cells	Electroconductive hydrogels	Spinal cord injury	Fan et al. (2022)
Gingival mesenchymal stem cells	Chitosan/Silk hydrogel sponge	Diabetic skin defects	Shi et al. (2017)
Induced pluripotent stem cell	Thermosensitive chitosan-based hydrogels	Corneal injury	Tang et al. (2022)

**TABLE 3:** Characteristics of different EVs and MSC lysates.

Vesicle type	Size	Origin	Function
Nanovesicles	8–12 nm	Currently unknown	cell–cell communication
Exosomes	30–150 nm	Released through MVB fusion with the plasma membrane	cell–cell communication
			Repair and regeneration of damaged tissue
			Regulation of he extracellular microenvironment
			May promote disease pathogenesis
Microvesicles	200–1,000 nm	Direct sprouting from the plasma membrane	cell–cell communication
			Repair and regeneration of damaged tissue
			Regulation of the extracellular microenvironment
			May promote disease pathogenesis
Apoptotic bodies	1,000–5,000 nm	Plasma membrane fragments of cytoplasm and organelles combined randomly	Rapid removal of cell debris
			Repair and regeneration of damaged tissue
			Potential drug-delivery systems
			Diagnostic tools
Large oncosomes	1–10 μm	Direct sprouting from the plasma membrane	cell–cell communication
			Regulation of the extracellular microenvironment
			May promote disease pathogenesis
MSC lysates	Full range of sizes	Repeated cell freeze–thaw cycles	Repair and regeneration of damaged tissue

In summary, although culture-condition stimulated exosomes or their loaded hydrogels have achieved remarkable success in several therapies and clinical trials, several challenges remain. According to the data and research results obtained to date, we would provide several suggestions as follows: 1) The combination of exosomes with hydrogels have been verified to facilitate tissue repair. Apart from enhancing biomaterials properties, how to deliver exosomes directly to target cells is more attractive. We could overcome this obstacle by focusing on extending the half-life and increasing the purity of exosomes. 2) The development of a normalized method for the collection and enrichment of exosomes is imperative to boost their production and therapeutic efficacy, we could refer to the International Society for Extracellular Vesicles (ISEV) 2018 guidelines to improve exosome production and purity. 3) Exosomes, EVs, and MSC lysates are theoretically superior to MSC in the context of regenerative medicine, however, various challenges and difficulties are needed to overcome to realize their therapeutic potential, comparing exosomes with other EVs and MSC lysates are needed to identify their strengths and weaknesses, and improve their application to scientific research, so further research should be done to provide references to overcome this hurdle.

## References

[B1] Akers J. C. Gonda D. Kim R. Carter B. S. Chen C. C. (2013). Biogenesis of extracellular vesicles (EV): Exosomes, microvesicles, retrovirus-like vesicles, and apoptotic bodies. J. Neurooncol. 113 (1), 1–11. 10.1007/s11060-013-1084-8 23456661 PMC5533094

[B2] Albashari A. He Y. Zhang Y. Ali J. Lin F. Zheng Z. (2020). Thermosensitive bFGF-modified hydrogel with dental pulp stem cells on neuroinflammation of spinal cord injury. ACS Omega 5 (26), 16064–16075. 10.1021/acsomega.0c01379 32656428 PMC7346236

[B3] Albersen M. Fandel T. M. Lin G. Wang G. Banie L. Lin C. S. (2010). Injections of adipose tissue-derived stem cells and stem cell lysate improve recovery of erectile function in a rat model of cavernous nerve injury. J. Sex. Med. 7 (10), 3331–3340. 10.1111/j.1743-6109.2010.01875.x 20561166 PMC3885341

[B4] Anderson J. D. Johansson H. J. Graham C. S. Vesterlund M. Pham M. T. Bramlett C. S. (2016). Comprehensive proteomic analysis of mesenchymal stem cell exosomes reveals modulation of angiogenesis via nuclear factor-KappaB signaling. Stem Cells 34 (3), 601–613. 10.1002/stem.2298 26782178 PMC5785927

[B5] Angelova P. R. Abramov A. Y. (2016). Functional role of mitochondrial reactive oxygen species in physiology. Free Radic. Biol. Med. 100, 81–85. 10.1016/j.freeradbiomed.2016.06.005 27296839

[B6] Atienzar-Aroca S. Flores-Bellver M. Serrano-Heras G. Martinez-Gil N. Barcia J. M. Aparicio S. (2016). Oxidative stress in retinal pigment epithelium cells increases exosome secretion and promotes angiogenesis in endothelial cells. J. Cell. Mol. Med. 20 (8), 1457–1466. 10.1111/jcmm.12834 26999719 PMC4956947

[B7] Atkin-Smith G. K. Miles M. A. Tixeira R. Lay F. T. Duan M. Hawkins C. J. (2019). Plexin B2 is a regulator of monocyte apoptotic cell disassembly. Cell Rep. 29 (7), 1821–1831.e3. e3. 10.1016/j.celrep.2019.10.014 31722200

[B8] Atkin-Smith G. K. Paone S. Zanker D. J. Duan M. Phan T. K. Chen W. (2017). Isolation of cell type-specific apoptotic bodies by fluorescence-activated cell sorting. Sci. Rep. 7, 39846. 10.1038/srep39846 28057919 PMC5216387

[B9] Azimi M. Ghabaee M. Naser Moghadasi A. Izad M. (2019). Altered expression of miR-326 in T cell-derived exosomes of patients with relapsing-remitting multiple sclerosis. Iran. J. Allergy Asthma Immunol. 18 (1), 108–113. 10.18502/ijaai.v18i1.636 30848579

[B10] Berchem G. Noman M. Z. Bosseler M. Paggetti J. Baconnais S. Le Cam E. (2015). Hypoxic tumor-derived microvesicles negatively regulate NK cell function by a mechanism involving TGF-β and miR23a transfer. Oncoimmunology 5 (4), e1062968. 10.1080/2162402X.2015.1062968 27141372 PMC4839360

[B11] Berniakovich I. Giorgio M. (2013). Low oxygen tension maintains multipotency, whereas normoxia increases differentiation of mouse bone marrow stromal cells. Int. J. Mol. Sci. 14 (1), 2119–2134. 10.3390/ijms14012119 23340651 PMC3565369

[B12] Blander J. M. (2017). The many ways tissue phagocytes respond to dying cells. Immunol. Rev. 277 (1), 158–173. 10.1111/imr.12537 28462530 PMC5721677

[B13] Brock C. K. Wallin S. T. Ruiz O. E. Samms K. M. Mandal A. Sumner E. A. (2019). Stem cell proliferation is induced by apoptotic bodies from dying cells during epithelial tissue maintenance. Nat. Commun. 10 (1), 1044. 10.1038/s41467-019-09010-6 30837472 PMC6400930

[B14] Butterfield D. A. Boyd-Kimball D. (2020). Mitochondrial oxidative and nitrosative stress and alzheimer disease. Antioxidants (Basel) 9 (9), 818. 10.3390/antiox9090818 PMC7554713 32887505

[B15] Camussi G. (2022). Exosomes and microvesicles: From stem cell biology to translation in human diseases. Stem Cell Rev. Rep. 18 (3), 853. 10.1007/s12015-022-10337-9 35089462

[B16] Cao J. Wang B. Tang T. Lv L. Ding Z. Li Z. (2020). Three-dimensional culture of MSCs produces exosomes with improved yield and enhanced therapeutic efficacy for cisplatin-induced acute kidney injury. Stem Cell Res. Ther. 11 (1), 206. 10.1186/s13287-020-01719-2 32460853 PMC7251891

[B17] Ceradini D. J. Kulkarni A. R. Callaghan M. J. Tepper O. M. Bastidas N. Kleinman M. E. (2004). Progenitor cell trafficking is regulated by hypoxic gradients through HIF-1 induction of SDF-1. Nat. Med. 10 (8), 858–864. 10.1038/nm1075 15235597

[B18] Chang Y. H. Wu K. C. Wang C. C. Ding D. C. (2020). Enhanced chondrogenesis of human umbilical cord mesenchymal stem cells in a gelatin honeycomb scaffold. J. Biomed. Mat. Res. A 108 (10), 2069–2079. 10.1002/jbm.a.36966 32323440

[B19] Chen P. Zheng L. Wang Y. Tao M. Xie Z. Xia C. (2019). Desktop-stereolithography 3D printing of a radially oriented extracellular matrix/mesenchymal stem cell exosome bioink for osteochondral defect regeneration. Theranostics 9 (9), 2439–2459. 10.7150/thno.31017 31131046 PMC6525998

[B20] Chen J. Chen J. Cheng Y. Fu Y. Zhao H. Tang M. (2020). Mesenchymal stem cell-derived exosomes protect beta cells against hypoxia-induced apoptosis via miR-21 by alleviating ER stress and inhibiting p38 MAPK phosphorylation. Stem Cell Res. Ther. 11 (1), 97. 10.1186/s13287-020-01610-0 32127037 PMC7055095

[B21] Chiaradia E. Tancini B. Emiliani C. Delo F. Pellegrino R. M. Tognoloni A. (2021). Extracellular vesicles under oxidative stress conditions: Biological properties and physiological roles. Cells 10 (7), 1763. 10.3390/cells10071763 34359933 PMC8306565

[B22] Clancy J. W. Sedgwick A. Rosse C. Muralidharan-Chari V. Raposo G. Method M. (2015). Regulated delivery of molecular cargo to invasive tumour-derived microvesicles. Nat. Commun. 6, 6919. 10.1038/ncomms7919 25897521 PMC4497525

[B23] Comelli L. Rocchiccioli S. Smirni S. Salvetti A. Signore G. Citti L. (2014). Characterization of secreted vesicles from vascular smooth muscle cells. Mol. Biosyst. 10 (5), 1146–1152. 10.1039/c3mb70544g 24626815

[B24] Conigliaro A. Costa V. Lo Dico A. Saieva L. Buccheri S. Dieli F. (2015). CD90+ liver cancer cells modulate endothelial cell phenotype through the release of exosomes containing H19 lncRNA. Mol. Cancer 14, 155. 10.1186/s12943-015-0426-x 26272696 PMC4536801

[B25] Crescitelli R. Lässer C. Szabó T. G. Kittel A. Eldh M. Dianzani I. (2013). Distinct RNA profiles in subpopulations of extracellular vesicles: Apoptotic bodies, microvesicles and exosomes. J. Extracell. Vesicles 2 (1), 20677. 10.3402/jev.v2i0.20677 PMC3823106 24223256

[B26] D'Souza-Schorey C. Clancy J. W. (2012). Tumor-derived microvesicles: Shedding light on novel microenvironment modulators and prospective cancer biomarkers. Genes Dev. 26 (12), 1287–1299. 10.1101/gad.192351.112 22713869 PMC3387656

[B27] Eldh M. Ekström K. Valadi H. Sjöstrand M. Olsson B. Jernås M. (2010). Exosomes communicate protective messages during oxidative stress; possible role of exosomal shuttle RNA. PLoS One 5 (12), e15353. 10.1371/journal.pone.0015353 21179422 PMC3003701

[B28] Fan L. Liu C. Chen X. Zheng L. Zou Y. Wen H. (2022). Exosomes-loaded electroconductive hydrogel synergistically promotes tissue repair after spinal cord injury via immunoregulation and enhancement of myelinated axon growth. Adv. Sci. (Weinh). 9 (13), e2105586. 10.1002/advs.202105586 35253394 PMC9069372

[B29] Fan Y. Shen B. Tan M. Mu X. Qin Y. Zhang F. (2014). Long non-coding RNA UCA1 increases chemoresistance of bladder cancer cells by regulating Wnt signaling. FEBS J. 281 (7), 1750–1758. 10.1111/febs.12737 24495014

[B30] Fu L. Wu S. S. (2021). Advances in studies on exosomes and microvesicles as markers of cardiovascular disease. Eur. Rev. Med. Pharmacol. Sci. 25 (6), 2622–2629. 10.26355/eurrev_202103_25426 33829449

[B31] Gao W. Liang T. He R. Ren J. Yao H. Wang K. (2020). Exosomes from 3D culture of marrow stem cells enhances endothelial cell proliferation, migration, and angiogenesis via activation of the HMGB1/AKT pathway. Stem Cell Res. 50, 102122. 10.1016/j.scr.2020.102122 33316600

[B32] Giri K. R. de Beaurepaire L. Jegou D. Lavy M. Mosser M. Dupont A. (2020). Molecular and functional diversity of distinct subpopulations of the stressed insulin-secreting cell's vesiculome. Front. Immunol. 11, 1814. 10.3389/fimmu.2020.01814 33101266 PMC7556286

[B33] Guan X. Avci-Adali M. Alarçin E. Cheng H. Kashaf S. S. Li Y. (2017). Development of hydrogels for regenerative engineering. Biotechnol. J. 12 (5), 1600394. 10.1002/biot.201600394 PMC5503693 28220995

[B34] Guan F. Xiang X. Xie Y. Li H. Zhang W. Shu Y. (2021). Simultaneous metabolomics and proteomics analysis of plasma-derived extracellular vesicles. Anal. Methods 13 (16), 1930–1938. 10.1039/d1ay00060h 33913941

[B35] Guo Y. Tan J. Miao Y. Sun Z. Zhang Q. (2019). Effects of microvesicles on cell apoptosis under hypoxia. Oxid. Med. Cell. Longev. 2019, 1–11. 10.1155/2019/5972152 PMC6501227 31178970

[B36] Gurunathan S. Kang M. H. Jeyaraj M. Qasim M. Kim J. H. (2019). Review of the isolation, characterization, biological function, and multifarious therapeutic approaches of exosomes. Cells 8 (4), 307. Erratum in: Cells. 2021 Feb 22;10(2). 10.3390/cells8040307 PMC6523673 30987213

[B37] Gurung S. Perocheau D. Touramanidou L. Baruteau J. (2021). The exosome journey: From biogenesis to uptake and intracellular signalling. Cell Commun. Signal. 19 (1), 47. 10.1186/s12964-021-00730-1 33892745 PMC8063428

[B38] Hade M. D. Suire C. N. Suo Z. (2021). Mesenchymal stem cell-derived exosomes: Applications in regenerative medicine. Cells 10 (8), 1959. 10.3390/cells10081959 34440728 PMC8393426

[B39] Hanson P. I. Cashikar A. (2012). Multivesicular body morphogenesis. Annu. Rev. Cell Dev. Biol. 28, 337–362. 10.1146/annurev-cellbio-092910-154152 22831642

[B40] Haraszti R. A. Miller R. Stoppato M. Sere Y. Y. Coles A. Didiot M. C. (2018). Exosomes produced from 3D cultures of MSCs by tangential flow filtration show higher yield and improved activity. Mol. Ther. 26 (12), 2838–2847. 10.1016/j.ymthe.2018.09.015 30341012 PMC6277553

[B41] Hashemi Tayer A. Amirizadeh N. Ahmadinejad M. Nikougoftar M. Deyhim M. R. Zolfaghari S. (2019). Procoagulant activity of red blood cell-derived microvesicles during red cell storage. Transfus. Med. Hemother. 46 (4), 224–230. 10.1159/000494367 31700504 PMC6739704

[B42] Hauser P. Wang S. Didenko V. V. (2017). Apoptotic bodies: Selective detection in extracellular vesicles. Methods Mol. Biol. 1554, 193–200. 10.1007/978-1-4939-6759-9_12 28185192

[B43] He C. Zheng S. Luo Y. Wang B. (2018). Exosome theranostics: Biology and translational medicine. Theranostics 8 (1), 237–255. 10.7150/thno.21945 29290805 PMC5743472

[B44] Hervera A. De Virgiliis F. Palmisano I. Zhou L. Tantardini E. Kong G. (2018). Reactive oxygen species regulate axonal regeneration through the release of exosomal NADPH oxidase 2 complexes into injured axons. Nat. Cell Biol. 20 (3), 307–319. 10.1038/s41556-018-0039-x 29434374

[B45] Hsu C. Morohashi Y. Yoshimura S. Manrique-Hoyos N. Jung S. Lauterbach M. A. (2010). Regulation of exosome secretion by Rab35 and its GTPase-activating proteins TBC1D10A-C. J. Cell Biol. 189 (2), 223–232. 10.1083/jcb.200911018 20404108 PMC2856897

[B46] Hsu M. F. Yu S. H. Chuang S. J. Kuo T. K. Singal P. K. Huang C. Y. (2018). Can mesenchymal stem cell lysate reverse aging? Aging (Albany NY) 10 (10), 2900–2910. 10.18632/aging.101595 30362957 PMC6224235

[B47] Hu X. Wu R. Shehadeh L. A. Zhou Q. Jiang C. Huang X. (2014). Severe hypoxia exerts parallel and cell-specific regulation of gene expression and alternative splicing in human mesenchymal stem cells. BMC Genomics 15, 303. 10.1186/1471-2164-15-303 24758227 PMC4234502

[B48] Huang J. Xiong J. Yang L. Zhang J. Sun S. Liang Y. (2021). Cell-free exosome-laden scaffolds for tissue repair. Nanoscale 13 (19), 8740–8750. 10.1039/d1nr01314a 33969373

[B49] Inal J. M. Ansa-Addo E. A. Stratton D. Kholia S. Antwi-Baffour S. S. Jorfi S. (2012). Microvesicles in health and disease. Arch. Immunol. Ther. Exp. Warsz. 60 (2), 107–121. 10.1007/s00005-012-0165-2 22307363

[B50] Jiang L. Tixeira R. Caruso S. Atkin-Smith G. K. Baxter A. A. Paone S. (2016). Monitoring the progression of cell death and the disassembly of dying cells by flow cytometry. Nat. Protoc. 11 (4), 655–663. 10.1038/nprot.2016.028 26938116

[B51] Jiang Y. Jiang H. Wang K. Liu C. Man X. Fu Q. (2021). Hypoxia enhances the production and antitumor effect of exosomes derived from natural killer cells. Ann. Transl. Med. 9 (6), 473. 10.21037/atm-21-347 33850870 PMC8039676

[B52] Juan T. Fürthauer M. (2018). Biogenesis and function of ESCRT-dependent extracellular vesicles. Semin. Cell Dev. Biol. 74, 66–77. 10.1016/j.semcdb.2017.08.022 28807885

[B53] Kamerkar S. LeBleu V. S. Sugimoto H. Yang S. Ruivo C. F. Melo S. A. (2017). Exosomes facilitate therapeutic targeting of oncogenic KRAS in pancreatic cancer. Nature 546 (7659), 498–503. 10.1038/nature22341 28607485 PMC5538883

[B54] Keshtkar S. Azarpira N. Ghahremani M. H. (2018). Mesenchymal stem cell-derived extracellular vesicles: Novel frontiers in regenerative medicine. Stem Cell Res. Ther. 9 (1), 63. 10.1186/s13287-018-0791-7 29523213 PMC5845209

[B55] Khayambashi P. Iyer J. Pillai S. Upadhyay A. Zhang Y. Tran S. D. (2021). Hydrogel encapsulation of mesenchymal stem cells and their derived exosomes for tissue engineering. Int. J. Mol. Sci. 22 (2), 684. 10.3390/ijms22020684 PMC7827932 33445616

[B56] Khubutiya M. S. Temnov A. A. Vagabov V. A. Sklifas A. N. Rogov K. A. Zhgutov Y. A. (2015). Effect of conditioned medium and bone marrow stem cell lysate on the course of acetaminophen-induced liver failure. Bull. Exp. Biol. Med. 159 (1), 118–123. 10.1007/s10517-015-2905-x 26033600

[B57] Kore R. A. Edmondson J. L. Jenkins S. V. Jamshidi-Parsian A. Dings R. P. M. Reyna N. S. (2018). Hypoxia-derived exosomes induce putative altered pathways in biosynthesis and ion regulatory channels in glioblastoma cells. Biochem. Biophys. Rep. 14, 104–113. 10.1016/j.bbrep.2018.03.008 29872742 PMC5986551

[B58] Kumar A. Deep G. (2020). Exosomes in hypoxia-induced remodeling of the tumor microenvironment. Cancer Lett. 488, 1–8. 10.1016/j.canlet.2020.05.018 32473240

[B59] Lee J. Y. Kim H. S. (2021). Extracellular vesicles in regenerative medicine: Potentials and challenges. Tissue Eng. Regen. Med. 18 (4), 479–484. 10.1007/s13770-021-00365-w 34297340 PMC8300067

[B60] Lee Y. El Andaloussi S. Wood M. J. (2012). Exosomes and microvesicles: Extracellular vesicles for genetic information transfer and gene therapy. Hum. Mol. Genet. 21 (R1), R125–R134. 10.1093/hmg/dds317 22872698

[B61] Li S. Luo L. He Y. Li R. Xiang Y. Xing Z. (2021). Dental pulp stem cell-derived exosomes alleviate cerebral ischaemia-reperfusion injury through suppressing inflammatory response. Cell Prolif. 54 (8), e13093. 10.1111/cpr.13093 34231932 PMC8349657

[B62] Li Y. Yang R. Guo B. Zhang H. Zhang H. Liu S. (2019). Exosomal miR-301 derived from mesenchymal stem cells protects myocardial infarction by inhibiting myocardial autophagy. Biochem. Biophys. Res. Commun. 514 (1), 323–328. 10.1016/j.bbrc.2019.04.138 31036323

[B63] Li S. Yuan L. Su L. Lian Z. Liu C. Zhang F. (2020). Decreased miR-92a-3p expression potentially mediates the pro-angiogenic effects of oxidative stress-activated endothelial cell-derived exosomes by targeting tissue factor. Int. J. Mol. Med. 46 (5), 1886–1898. 10.3892/ijmm.2020.4713 32901851 PMC7521555

[B64] Liang Z. Luo Y. Lv Y. (2020). Mesenchymal stem cell-derived microvesicles mediate BMP2 gene delivery and enhance bone regeneration. J. Mat. Chem. B 8 (30), 6378–6389. 10.1039/d0tb00422g 32633309

[B65] Liao C. F. Lin S. H. Chen H. C. Tai C. J. Chang C. C. Li L. T. (2012). CSE1L, a novel microvesicle membrane protein, mediates Ras-triggered microvesicle generation and metastasis of tumor cells. Mol. Med. 18 (1), 1269–1280. 10.2119/molmed.2012.00205 22952058 PMC3521793

[B66] Luo L. Albashari A. A. Wang X. Jin L. Zhang Y. Zheng L. (2018). Effects of transplanted heparin-poloxamer hydrogel combining dental pulp stem cells and bFGF on spinal cord injury repair. Stem Cells Int. 2018, 2398521. 10.1155/2018/2398521 29765407 PMC5892218

[B67] Liu J. Qiu X. Lv Y. Zheng C. Dong Y. Dou G. (2020). Apoptotic bodies derived from mesenchymal stem cells promote cutaneous wound healing via regulating the functions of macrophages. Stem Cell Res. Ther. 11 (1), 507. 10.1186/s13287-020-02014-w 33246491 PMC7694913

[B68] Liu H. Liu S. Qiu X. Yang X. Bao L. Pu F. (2020). Donor MSCs release apoptotic bodies to improve myocardial infarction via autophagy regulation in recipient cells. Autophagy 16 (12), 2140–2155. 10.1080/15548627.2020.1717128 31959090 PMC7751634

[B69] Liu W. Li L. Rong Y. Qian D. Chen J. Zhou Z. (2020). Hypoxic mesenchymal stem cell-derived exosomes promote bone fracture healing by the transfer of miR-126. Acta Biomater. 103, 196–212. 10.1016/j.actbio.2019.12.020 31857259

[B70] Ma Q. Liang M. Wu Y. Ding N. Duan L. Yu T. (2019). Mature osteoclast-derived apoptotic bodies promote osteogenic differentiation via RANKL-mediated reverse signaling. J. Biol. Chem. 294 (29), 11240–11247. 10.1074/jbc.RA119.007625 31167789 PMC6643026

[B71] Ma Z. J. Yang J. J. Lu Y. B. Liu Z. Y. Wang X. X. (2020). Mesenchymal stem cell-derived exosomes: Toward cell-free therapeutic strategies in regenerative medicine. World J. Stem Cells 12 (8), 814–840. 10.4252/wjsc.v12.i8.814 32952861 PMC7477653

[B72] Madrigal M. Rao K. S. Riordan N. H. (2014). A review of therapeutic effects of mesenchymal stem cell secretions and induction of secretory modification by different culture methods. J. Transl. Med. 12, 260. 10.1186/s12967-014-0260-8 25304688 PMC4197270

[B73] Mao G. Xu Y. Long D. Sun H. Li H. Xin R. (2021). Exosome-transported circRNA_0001236 enhances chondrogenesis and suppress cartilage degradation via the miR-3677-3p/Sox9 axis. Stem Cell Res. Ther. 12 (1), 389. 10.1186/s13287-021-02431-5 34256841 PMC8278601

[B74] McMahon H. T. Boucrot E. (2015). Membrane curvature at a glance. J. Cell Sci. 128 (6), 1065–1070. 10.1242/jcs.114454 25774051 PMC4359918

[B75] Miao C. Zhou W. Wang X. Fang J. (2021). The research progress of exosomes in osteoarthritis, with particular emphasis on the mediating roles of miRNAs and lncRNAs. Front. Pharmacol. 12, 685623. 10.3389/fphar.2021.685623 34093208 PMC8176107

[B76] Mohyeldin A. Garzón-Muvdi T. Quiñones-Hinojosa A. (2010). Oxygen in stem cell biology: A critical component of the stem cell niche. Cell Stem Cell 7 (2), 150–161. 10.1016/j.stem.2010.07.007 20682444

[B77] Moldogazieva N. T. Mokhosoev I. M. Feldman N. B. Lutsenko S. V. (2018). ROS and RNS signalling: Adaptive redox switches through oxidative/nitrosative protein modifications. Free Radic. Res. 52 (5), 507–543. 10.1080/10715762.2018.1457217 29589770

[B78] Muralidharan-Chari V. Clancy J. Plou C. Romao M. Chavrier P. Raposo G. (2009). ARF6-regulated shedding of tumor cell-derived plasma membrane microvesicles. Curr. Biol. 19 (22), 1875–1885. 10.1016/j.cub.2009.09.059 19896381 PMC3150487

[B79] Nagata S. (2018). Apoptosis and clearance of apoptotic cells. Annu. Rev. Immunol. 36, 489–517. 10.1146/annurev-immunol-042617-053010 29400998

[B80] Namazi H. Mohit E. Namazi I. Rajabi S. Samadian A. Hajizadeh-Saffar E. (2018). Exosomes secreted by hypoxic cardiosphere-derived cells enhance tube formation and increase pro-angiogenic miRNA. J. Cell. Biochem. 119 (5), 4150–4160. 10.1002/jcb.26621 29243842

[B81] Namazi H. Namazi I. Ghiasi P. Ansari H. Rajabi S. Hajizadeh-Saffar E. (2018). Exosomes secreted by normoxic and hypoxic cardiosphere-derived cells have anti-apoptotic effect. Iran. J. Pharm. Res. 17 (1), 377–385. 10.18502/ijaai.v18i1.636 29755568 PMC5937107

[B82] Nassar W. El-Ansary M. Sabry D. Mostafa M. A. Fayad T. Kotb E. (2016). Umbilical cord mesenchymal stem cells derived extracellular vesicles can safely ameliorate the progression of chronic kidney diseases. Biomater. Res. 20, 21. 10.1186/s40824-016-0068-0 27499886 PMC4974791

[B83] Nikfarjam S. Rezaie J. Zolbanin N. M. Jafari R. (2020). Mesenchymal stem cell derived-exosomes: A modern approach in translational medicine. J. Transl. Med. 18 (1), 449. 10.1186/s12967-020-02622-3 33246476 PMC7691969

[B84] Nishikawa T. Maeda K. Nakamura M. Yamamura T. Sawada T. Mizutani Y. (2021). Filtrated adipose tissue-derived mesenchymal stem cell lysate ameliorates experimental acute colitis in mice. Dig. Dis. Sci. 66 (4), 1034–1044. 10.1007/s10620-020-06359-3 32488819

[B85] Nojehdehi S. Soudi S. Hesampour A. Rasouli S. Soleimani M. Hashemi S. M. (2018). Immunomodulatory effects of mesenchymal stem cell-derived exosomes on experimental type-1 autoimmune diabetes. J. Cell. Biochem. 119 (11), 9433–9443. 10.1002/jcb.27260 30074271

[B86] Nyam-Erdene A. Nebie O. Delila L. Buée L. Devos D. Chou S. Y. (2021). Characterization and chromatographic isolation of platelet extracellular vesicles from human platelet lysates for applications in neuroregenerative medicine. ACS Biomater. Sci. Eng. 7 (12), 5823–5835. 10.1021/acsbiomaterials.1c01226 34846835

[B87] Ostrowski M. Carmo N. B. Krumeich S. Fanget I. Raposo G. Savina A. (2010). Rab27a and Rab27b control different steps of the exosome secretion pathway. Nat. Cell Biol. 12 (1), 19–30. 10.1038/ncb2000 19966785

[B88] Palma M. Hansson L. Choudhury A. Näsman-Glaser B. Eriksson I. Adamson L. (2012). Vaccination with dendritic cells loaded with tumor apoptotic bodies (Apo-DC) in patients with chronic lymphocytic leukemia: Effects of various adjuvants and definition of immune response criteria. Cancer Immunol. Immunother. 61 (6), 865–879. 10.1007/s00262-011-1149-5 22086161 PMC11029556

[B89] Pant T. Juric M. Bosnjak Z. J. Dhanasekaran A. (2021). Recent insight on the non-coding RNAs in mesenchymal stem cell-derived exosomes: Regulatory and therapeutic role in regenerative medicine and tissue engineering. Front. Cardiovasc. Med. 8, 737512. 10.3389/fcvm.2021.737512 34660740 PMC8517144

[B90] Pegtel D. M. Gould S. J. (2019). Annu. Rev. Biochem. 88, 487–514. 10.1146/annurev-biochem-013118-111902 31220978

[B91] Pekarev O. G. Pekareva E. O. Mayborodin I. V. Silachev D. N. Baranov II Pozdnyakov I. M. (2021). The potential of extracellular microvesicles of mesenchymal stromal cells in obstetrics. J. Matern. Fetal. Neonatal Med., 1–3. 10.1080/14767058.2021.1951213 34344283

[B92] Poon I. K. Lucas C. D. Rossi A. G. Ravichandran K. S. (2014). Apoptotic cell clearance: Basic biology and therapeutic potential. Nat. Rev. Immunol. 14 (3), 166–180. 10.1038/nri3607 24481336 PMC4040260

[B93] Rahimi B. Panahi M. Saraygord-Afshari N. Taheri N. Bilici M. Jafari D. (2021). The secretome of mesenchymal stem cells and oxidative stress: Challenges and opportunities in cell-free regenerative medicine. Mol. Biol. Rep. 48 (7), 5607–5619. 10.1007/s11033-021-06360-7 34191238

[B94] Ratajczak M. Z. Ratajczak J. (2020). Extracellular microvesicles/exosomes: Discovery, disbelief, acceptance, and the future? Leukemia 34 (12), 3126–3135. 10.1038/s41375-020-01041-z 32929129 PMC7685969

[B95] Rezaie J. Ajezi S. Avci Ç. B. Karimipour M. Geranmayeh M. H. Nourazarian A. (2018). Exosomes and their application in biomedical field: Difficulties and advantages. Mol. Neurobiol. 55 (4), 3372–3393. 10.1007/s12035-017-0582-7 28497202

[B96] Saeed-Zidane M. Linden L. Salilew-Wondim D. Held E. Neuhoff C. Tholen E. (2017). Cellular and exosome mediated molecular defense mechanism in bovine granulosa cells exposed to oxidative stress. PLoS One 12 (11), e0187569. 10.1371/journal.pone.0187569 29117219 PMC5678720

[B97] Safari B. Aghazadeh M. Davaran S. Roshangar L. (2022). Exosome-loaded hydrogels: A new cell-free therapeutic approach for skin regeneration. Eur. J. Pharm. Biopharm. 171, 50–59. 10.1016/j.ejpb.2021.11.002 34793943

[B98] Santavanond J. P. Rutter S. F. Atkin-Smith G. K. Poon I. K. H. (2021). Apoptotic bodies: Mechanism of formation, isolation and functional relevance. Subcell. Biochem. 97, 61–88. 10.1007/978-3-030-67171-6_4 33779914

[B99] Savina A. Vidal M. Colombo M. I. (2002). The exosome pathway in K562 cells is regulated by Rab11. J. Cell Sci. 115 (12), 2505–2515. 10.1242/jcs.115.12.2505 12045221

[B100] Sedgwick A. E. Clancy J. W. Olivia Balmert M. D'Souza-Schorey C. (2015). Extracellular microvesicles and invadopodia mediate non-overlapping modes of tumor cell invasion. Sci. Rep. 5, 14748. 10.1038/srep14748 26458510 PMC4602187

[B101] Sedgwick A. E. D'Souza-Schorey C. (2018). The biology of extracellular microvesicles. Traffic 19 (5), 319–327. 10.1111/tra.12558 29479795 PMC6922305

[B102] Shafiei M. Ansari M. N. M. Razak S. I. A. Khan M. U. A. (2021). A comprehensive review on the applications of exosomes and liposomes in regenerative medicine and tissue engineering. Polym. (Basel) 13 (15), 2529. 10.3390/polym13152529 PMC8347192 34372132

[B103] Shahin H. I. Radnaa E. Tantengco O. A. G. Kechichian T. Kammala A. K. Sheller-Miller S. (2021). Microvesicles and exosomes released by amnion epithelial cells under oxidative stress cause inflammatory changes in uterine cells. Biol. Reprod. 105 (2), 464–480. 10.1093/biolre/ioab088 33962471 PMC8335356

[B104] Shen B. Liu J. Zhang F. Wang Y. Qin Y. Zhou Z. (2016). CCR2 positive exosome released by mesenchymal stem cells suppresses macrophage functions and alleviates ischemia/reperfusion-induced renal injury. Stem Cells Int. 2016, 1240301–1240309. 10.1155/2016/1240301 27843457 PMC5098097

[B105] Shi Q. Qian Z. Liu D. Sun J. Wang X. Liu H. (2017). GMSC-derived exosomes combined with a chitosan/silk hydrogel sponge accelerates wound healing in a diabetic rat skin defect model. Front. Physiol. 8, 904. 10.3389/fphys.2017.00904 29163228 PMC5681946

[B106] Shi M. M. Yang Q. Y. Monsel A. Yan J. Y. Dai C. X. Zhao J. Y. (2021). Preclinical efficacy and clinical safety of clinical-grade nebulized allogenic adipose mesenchymal stromal cells-derived extracellular vesicles. J. Extracell. Vesicles 10 (10), e12134. 10.1002/jev2.12134 34429860 PMC8363910

[B107] Shifrin D. A. Jr Demory Beckler M. Coffey R. J. Tyska M. J. (2013). Extracellular vesicles: Communication, coercion, and conditioning. Mol. Biol. Cell 24 (9), 1253–1259. 10.1091/mbc.E12-08-0572 23630232 PMC3639038

[B108] Shigemoto-Kuroda T. Oh J. Y. Kim D. K. Jeong H. J. Park S. Y. Lee H. J. (2017). MSC-Derived extracellular vesicles attenuate immune responses in two autoimmune murine models: Type 1 diabetes and uveoretinitis. Stem Cell Rep. 8 (5), 1214–1225. 10.1016/j.stemcr.2017.04.008 PMC5425726 28494937

[B109] Sies H. Berndt C. Jones D. P. (2017). Oxidative stress. Annu. Rev. Biochem. 86, 715–748. 10.1146/annurev-biochem-061516-045037 28441057

[B110] Slaughter B. V. Khurshid S. S. Fisher O. Z. Khademhosseini A. Peppas N. A. (2009). Hydrogels in regenerative medicine. Adv. Mat. 21 (32-33), 3307–3329. 10.1002/adma.200802106 PMC4494665 20882499

[B111] Ståhl A. L. Johansson K. Mossberg M. Kahn R. Karpman D. (2019). Exosomes and microvesicles in normal physiology, pathophysiology, and renal diseases. Pediatr. Nephrol. 34 (1), 11–30. 10.1007/s00467-017-3816-z 29181712 PMC6244861

[B112] Sun X. Meng H. Wan W. Xie M. Wen C. (2019). Application potential of stem/progenitor cell-derived extracellular vesicles in renal diseases. Stem Cell Res. Ther. 10 (1), 8. 10.1186/s13287-018-1097-5 30616603 PMC6323814

[B113] Suzuki J. Fujii T. Imao T. Ishihara K. Kuba H. Nagata S. (2013). Calcium-dependent phospholipid scramblase activity of TMEM16 protein family members. J. Biol. Chem. 288 (19), 13305–13316. 10.1074/jbc.M113.457937 23532839 PMC3650369

[B114] Takasugi M. Okada R. Takahashi A. Virya Chen D. Watanabe S. Hara E. (2017). Small extracellular vesicles secreted from senescent cells promote cancer cell proliferation through EphA2. Nat. Commun. 8, 15729. 10.1038/ncomms15728 PMC5467215 28585531

[B115] Tang Q. Lu B. He J. Chen X. Fu Q. Han H. (2022). Exosomes-loaded thermosensitive hydrogels for corneal epithelium and stroma regeneration. Biomaterials 280, 121320. 10.1016/j.biomaterials.2021.121320 34923312

[B116] Tang Y. Zhou Y. Li H. J. (2021). Advances in mesenchymal stem cell exosomes: A review. Stem Cell Res. Ther. 12 (1), 71. 10.1186/s13287-021-02138-7 33468232 PMC7814175

[B117] Tricarico C. Clancy J. D'Souza-Schorey C. (2017). Biology and biogenesis of shed microvesicles. Small GTPases 8 (4), 220–232. 10.1080/21541248.2016.1215283 27494381 PMC5680703

[B118] Wang X. S. Zhang Z. Wang H. C. Cai J. L. Xu Q. W. Li M. Q. (2006). Rapid identification of UCA1 as a very sensitive and specific unique marker for human bladder carcinoma. Clin. Cancer Res. 12 (16), 4851–4858. 10.1158/1078-0432.CCR-06-0134 16914571

[B119] Wang F. Li X. Xie X. Zhao L. Chen W. (2008). UCA1, a non-protein-coding RNA up-regulated in bladder carcinoma and embryo, influencing cell growth and promoting invasion. FEBS Lett. 582 (13), 1919–1927. 10.1016/j.febslet.2008.05.012 18501714

[B120] Wang T. Gilkes D. M. Takano N. Xiang L. Luo W. Bishop C. J. (2014). Hypoxia-inducible factors and RAB22A mediate formation of microvesicles that stimulate breast cancer invasion and metastasis. Proc. Natl. Acad. Sci. U. S. A. 111 (31), E3234–E3242. 10.1073/pnas.1410041111 24938788 PMC4128139

[B121] Wang L. Gu Z. Zhao X. Yang N. Wang F. Deng A. (2016). Extracellular vesicles released from human umbilical cord-derived mesenchymal stromal cells prevent life-threatening acute graft-versus-host disease in a mouse model of allogeneic hematopoietic stem cell transplantation. Stem Cells Dev. 25 (24), 1874–1883. 10.1089/scd.2016.0107 27649744

[B122] Wang Q. He Y. Zhao Y. Xie H. Lin Q. He Z. (2017). A thermosensitive heparin-poloxamer hydrogel bridges aFGF to treat spinal cord injury. ACS Appl. Mat. Interfaces 9 (8), 6725–6745. 10.1021/acsami.6b13155 28181797

[B123] Wang G. Yuan J. Cai X. Xu Z. Wang J. Ocansey D. K. W. (2020). HucMSC-exosomes carrying miR-326 inhibit neddylation to relieve inflammatory bowel disease in mice. Clin. Transl. Med. 10 (2), e113. 10.1002/ctm2.113 32564521 PMC7403704

[B124] Wang Y. (2018). Programmable hydrogels. Biomaterials 178, 663–680. 10.1016/j.biomaterials.2018.03.008 29549970 PMC6054804

[B125] Xin H. Li Y. Liu Z. Wang X. Shang X. Cui Y. (2013). MiR-133b promotes neural plasticity and functional recovery after treatment of stroke with multipotent mesenchymal stromal cells in rats via transfer of exosome-enriched extracellular particles. Stem Cells 31 (12), 2737–2746. 10.1002/stem.1409 23630198 PMC3788061

[B126] Xue M. Chen W. Xiang A. Wang R. Chen H. Pan J. (2017). Hypoxic exosomes facilitate bladder tumor growth and development through transferring long non-coding RNA-UCA1. Mol. Cancer 16 (1), 143. 10.1186/s12943-017-0714-8 28841829 PMC5574139

[B127] Xu X. Lai Y. Hua Z. C. (2019). Apoptosis and apoptotic body: Disease message and therapeutic target potentials. Biosci. Rep. 39 (1), BSR20180992. 10.1042/BSR20180992 30530866 PMC6340950

[B128] Yan L. Wu X. (2020). Exosomes produced from 3D cultures of umbilical cord mesenchymal stem cells in a hollow-fiber bioreactor show improved osteochondral regeneration activity. Cell Biol. Toxicol. 36 (2), 165–178. 10.1007/s10565-019-09504-5 31820164 PMC7196084

[B129] Yang S. Zhu B. Yin P. Zhao L. Wang Y. Fu Z. (2020). Integration of human umbilical cord mesenchymal stem cells-derived exosomes with hydroxyapatite-embedded hyaluronic acid-alginate hydrogel for bone regeneration. ACS Biomater. Sci. Eng. 6 (3), 1590–1602. 10.1021/acsbiomaterials.9b01363 33455380

[B130] Yang L. Zhai Y. Hao Y. Zhu Z. Cheng G. (2020). The regulatory functionality of exosomes derived from hUMSCs in 3D culture for alzheimer's disease therapy. Small 16 (3), e1906273. 10.1002/smll.201906273 31840420

[B131] Ye Q. Zhang Y. S. (2022). The era of translational nanomedicine. Nano TransMed 1 (1), e9130006. 10.26599/NTM.2022.9130006

[B132] Zhang Y. Chopp M. Zhang Z. G. Katakowski M. Xin H. Qu C. (2017). Systemic administration of cell-free exosomes generated by human bone marrow derived mesenchymal stem cells cultured under 2D and 3D conditions improves functional recovery in rats after traumatic brain injury. Neurochem. Int. 111, 69–81. 10.1016/j.neuint.2016.08.003 27539657 PMC5311054

[B133] Zhang K. Zhao X. Chen X. Wei Y. Du W. Wang Y. (2018). Enhanced therapeutic effects of mesenchymal stem cell-derived exosomes with an injectable hydrogel for hindlimb ischemia treatment. ACS Appl. Mat. Interfaces 10 (36), 30081–30091. 10.1021/acsami.8b08449 30118197

[B134] Zhang Q. Huang X. M. Liao J. X. Dong Y. K. Zhu J. L. He C. C. (2021). LncRNA HOTAIR promotes neuronal damage through facilitating NLRP3 mediated-pyroptosis activation in Parkinson's disease via regulation of miR-326/ELAVL1 Axis. Cell. Mol. Neurobiol. 41 (8), 1773–1786. 10.1007/s10571-020-00946-8 32968928 PMC11444004

[B135] Zhang T. Gaffrey M. J. Li X. Qian W. J. (2021). Characterization of cellular oxidative stress response by stoichiometric redox proteomics. Am. J. Physiology-Cell Physiology 320 (2), C182–C194. 10.1152/ajpcell.00040.2020 PMC7948008 33264075

[B136] Zhu J. Lu K. Zhang N. Zhao Y. Ma Q. Shen J. (2018). Myocardial reparative functions of exosomes from mesenchymal stem cells are enhanced by hypoxia treatment of the cells via transferring microRNA-210 in an nSMase2-dependent way. Artif. Cells Nanomed. Biotechnol. 46 (8), 1659–1670. 10.1080/21691401.2017.1388249 29141446 PMC5955787

[B137] Zhu Y. G. Shi M. M. Monsel A. Dai C. X. Dong X. Shen H. (2022). Nebulized exosomes derived from allogenic adipose tissue mesenchymal stromal cells in patients with severe COVID-19: A pilot study. Stem Cell Res. Ther. 13 (1), 220. 10.1186/s13287-022-02900-5 35619189 PMC9135389

